# An intronic variant in the *GCKR* gene is associated with multiple lipids

**DOI:** 10.1038/s41598-019-46750-3

**Published:** 2019-07-15

**Authors:** Lilian Fernandes Silva, Jagadish Vangipurapu, Teemu Kuulasmaa, Markku Laakso

**Affiliations:** 1 Institute of Clinical Medicine, Internal Medicine, University of Eastern Finland, Kuopio, Finland; 2 Department of Medicine, Kuopio University Hospital, Kuopio, Finland

**Keywords:** Diabetes complications, Genetic association study

## Abstract

Previous studies have shown that an intronic variant rs780094 of the *GCKR* gene (glucokinase regulatory protein) is significantly associated with several metabolites, but the associations of this genetic variant with different lipids is largely unknown. Therefore, we applied metabolomics approach to measure metabolites in a large Finnish population sample (METSIM study) to investigate their associations with rs780094 of *GCKR*. We measured metabolites by mass spectrometry from 5,181 participants. *P* < 5.8 × 10^−5^ was considered as statistically significant given 857 metabolites included in statistical analyses. We found novel negative associations of the T allele of *GCKR* rs780094 with serine and threonine, and positive associations with two metabolites of tryptophan, indolelactate and N-acetyltryptophan. Additionally, we found novel significant positive associations of this genetic variant with 12 glycerolipids and 19 glycerophospholipids. Significant negative associations were found for three glycerophospholipids (all plasmalogen-cholines), and two sphingolipids. Significant novel associations were also found with gamma-glutamylthreonine, taurocholenate sulfate, and retinol. Our study adds new information about the pleiotropy of the *GCKR* gene, and shows the associations of the T allele of *GCKR* rs780094 with lipids.

## Introduction

Genetic and lifestyle/environmental factors play an important role in the risk of type 2 diabetes. Over 100 genetic variants have been associated with type 2 diabetes in recent genome wide association studies, including glucokinase regulator *GCKR* gene encoding glucokinase regulatory protein (GKRP), a hepatocyte-specific inhibitor of the glucose-metabolizing enzyme glucokinase in the fasting state. After a meal hepatic glucokinase is released to cytoplasm and stimulates glycogen deposition and *de novo* lipogenesis^1^.

Two single nucleotide polymorphisms of *GCKR*, rs780094 (intronic variant) and rs1260326 (p.P446L) are in a strong linkage disequilibrium. These genetic variants are significantly associated with several metabolites, including amino acids, carbohydrates, ketone bodies, high density lipoprotein, total triacylglycerides (TAG), very low density lipoprotein, and diseases, such as type 2 diabetes, non-alcoholic fatty liver disease (NAFLD) and nonalcoholic steatohepatitis (NASH)^2–7^. Thus, *GCKR* is a highly pleiotropic gene. The p.P446L GKRP substitution results in destabilization of the GCK binding interface explaining inverse correlation between fasting glucose and triglycerides for this variant^8^. Increased hepatic GCK activity results in decreased glucose levels and increases in TAG and glycogen synthesis in normoglycemia^9^.

Previous studies on the associations of genetic variants of *GCKR* with laboratory measurements have not focused on lipids except for TAG. Given the fact that variants of *GCKR* have been associated with liver diseases the associations of these variants with lipids, including glycerolipids (GLs), glycerophospholipids (GPLs) and sphingolipids could be of great interest. GLs are composed of mono, di-, and tri-substituted glycerols that are hydrolyzed to free fatty acids (FFAs)^10^. GPLs are major components of cellular membranes synthesized from phosphatidic acid (PA) and diacylglycerol (DAG)^11^. PA can also be formed *via* phosphorylation of DAG^11^ and dephosphorylated to generate 1,2-DAG or bound with choline, ethanolamine or inositol to synthesize phosphatidylcholine (PC), phosphatidylethanolamine (PE), and phosphatidylinositol (PI)^11^.

We applied metabolomics to measure low molecular weight molecules in the Finnish prospective population-based Metabolic Syndrome In Men (METSIM) study to obtain a comprehensive understanding of metabolites associated with rs780094 of *GCKR*^12^. A special focus in our study is on the associations of this genetic variant with different lipids because they play an important role in liver disease, especially in NAFLD.

## Results

### Principal component analysis and correlations

We performed the principal component analysis (PCA) for the lipids. As shown in Supplementary Table S1, 33 metabolites resulted in 7 components explaining 77.2% of total variance. The first principal component (PC) having the largest loading for oleoyl-linoleoyl-glycerol (18:1/18:2)2 and other glycerolipids explained 38.5% of total variance. The second PCs had the highest loading for 1-stearoyl-2-linoleoyl-GPE (18:0/18:2) and explained 12.6%, and the third PC having the highest loading for 1-myristoyl-2-palmitoyl-GPC (14:0/16:0) explained 8.0% of total variance. The second and third PCs had high loadings for glycerophospholipids. These three PCs explained 59.1% of total variance.

The heat map shows the correlations between the lipids (Supplementary Fig. S1). Glycerolipids (the lower left corner of Supplementary Fig. S1) were loaded mainly on the first PC (PC1). Glycerophospholipids were loaded on both PC2 and PC3, and had high intercorrelations. Sphingolipids were mainly loaded on PC4 (Supplementary Table S1) and had no correlation or negative correlations with all other lipids demonstrating the heterogeneity among lipid species. The strongest correlation (r = 0.840) was observed between the two isomers of oleoyl-arachidonoyl-glycerol (18:1/20:4)1 and oleoyl-arachidonoyl-glycerol (18:1/20:4)2. Lipids and carbohydrates were also significantly correlated, 1-stearoyl-2-oleoyl-GPE (18:0/18:1) and pyruvate had a correlation of 0.358, as well as lipids and amino acids, 1-myristoyl-2-arachidonoyl-GPC (14:0/20:4) and alanine had a correlation of 0.287.

### Association of the T allele of *GCKR* rs780094 with amino acids, carbohydrates, and other metabolites

After adjustment for batch effect, age and fasting glucose *GCKR* rs780094-T showed a significant positive association (*P* < 5.8 × 10^−5^) with alanine (*P* = 7.9 × 10^−10^), and downstream metabolites of the tryptophan pathway metabolites, indolelactate and N-acetyltryptophan (*P* = 2.1 × 10^−6^, and *P* = 5.2 × 10^−7^, respectively), and novel negative associations with serine (*P* = 2.1 × 10^−6^), threonine (*P* = 4.8 × 10^−11^), and 3-aminoisobutyrate (*P* = 3.5 × 10^−10^) which is a downstream metabolite of the valine pathway Table 1. We also found significant associations of *GCKR* rs780094-T with lactate, pyruvate and mannose, as previously published. Novel negative associations were also found with gamma-glutamylthreonine (*P* = 4.9 × 10^−6^), taurocholenate sulfate (*P* = 8.1 × 10^−6^), and a novel positive association with retinol (*P* = 4.8 × 10^−6^) (Table 1). Nominally significant associations (*P* < 0.05) of *GCKR* rs780094-T with the metabolites are shown in Supplementary Table S2, and non-significant associations of *GCKR* rs780094-T with metabolites in Supplementary Table S3.

**Table 1:** Associations of *GCKR* rs780094-T with amino acids, carbohydrates and other metabolites.

Pathway	beta	*P* value	P* value	Novel
**Amino acids**				
Alanine	0.080	1.3E-08	7.9E10-10	No
Serine	−0.065	3.5E-06	2.1E10-6	Yes
Threonine	−0.094	1.8E-11	4.8E10-11	Yes
**Tryptophan pathway:**				
Indolelactate	0.066	2.1E-06	2.1E10-6	Yes
N-acetyltryptophan	0.066	2.1E-06	5.2E10-7	Yes
Valine pathway:				
3-aminoisobutyrate	−0.082	4.1E-09	3.5E10-10	Yes
**Carbohydrates**				
Lactate	0.066	2.6E-06	3.5E10-7	No
Mannose	−0.325	1.8E-125	8.5E10-128	No
Pyruvate	0.083	2.9E-09	1.4E10-10	No
**Other metabolites**				
Gamma-glutamylthreonine	−0.065	3.3E-06	4.9E10-6	Yes
Taurocholenate sulfate	−0.063	6.7E-06	8.1E10-6	Yes
Retinol (Vitamin A)	0.063	6.6E-06	4.8E10-7	Yes
3-hydroxybutyrate	−0.057	4.7E-05	1.4E10-5	No

Beta and *P* values were obtained from linear regression and adjusted for batch effect. Only metabolites that were associated significantly (*P* < 5.8 × 10^−5^) are shown. Beta and *P* values were obtained from linear regression, and were adjusted for batch effect, P* values were adjusted for batch effect, age and fasting glucose.

### Association of rs780094 of GCKR with lipids

#### Glycerolipids

**Figure 1:**
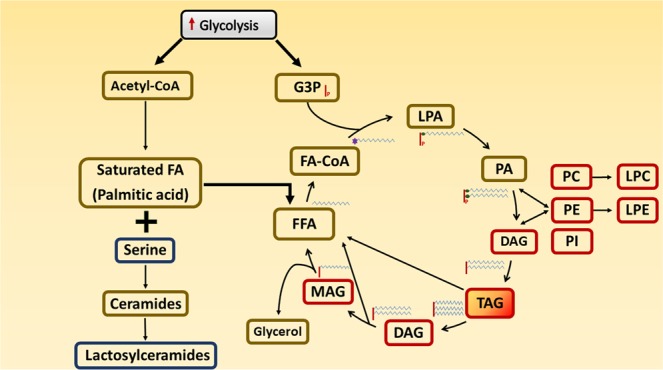
Increased levels of metabolites in the lipid pathways in carriers of *GCKR* rs780094-T are marked by red frames, decreased metabolite levels by blue frames, and other metabolites by brown frames. Increased glycolysis stimulates the formation of glycerol-3-phosphate (G3P) and acetyl coenzyme A (acetyl-CoA). G3P conjugates with fatty acyl coenzyme A (FA-CoA) to generate LPA (lysophosphatidic acid) and phosphatidic acid (PA), which is the precursor for all glycerolipids (GLs) and glycerophospholipids (GPLs). GLs are composed of mono- (MAG), di- (DAG), and trisubstituted glycerols (TAG) that are hydrolyzed to free fatty acids (FFAs). GPLs produce phosphatidic acid (PA) and DAG. PA can also be formed *via* phosphorylation of DAG, and dephosphorylated to generate 1,2-DAG or bound with choline, ethanolamine or inositol to synthetize phosphatidylcholine (PC), phosphatidylethanolamine (PE), and phosphatidylinositol (PI). Increased glycolysis and low serine level enhance *de novo* lipogenesis and the FFA/GL cycling. Acetyl-CoA initiates the *de novo* lipogenesis pathway by generating saturated fatty acids, such as palmitic acid and monounsaturated fatty acid, oleic acid. The excess of palmitic acid is directed to the FFA/GL cycling by unsaturated fatty acids, such as oleate, and leads to increasing levels of DAG, TAG and GPLs, resulting in fat accumulation in the liver. Low availability of serine leads to an increase in palmitic acid levels, and a decrease in ceramide and lactosylceramide levels. LPC, Lysophosphatidylcholine; LPE, Lysophosphatidylethanolamine.

We found 12 novel significant positive associations of *GCKR* rs780094-T with GLs after the adjustment for batch effect, age and fasting glucose. Nine of them were 1,2-DAGs and three monoacylglycerides Table 2, Fig. 1.

**Table 2:** Association of *GCKR* rs780094-T with lipids.

Pathway	Beta	*P* value	P* value	Sub class	Direct parent	Novel
**Glycerolipids**						
Triacylglycerides*	0.107	2.1E-14	1.6E10-16	TAG	TAG	No
Palmitoleoyl-linoleoyl-glycerol (16:1/18:2) (1)**	0.109	3.8E-09	6.9E10-10	DAG	1,2-DAG	Yes
Myristoyl-linoleoyl-glycerol (14:0/18:2) (1)**	0.120	7.4E-11	2.2E10-11	DAG	1,2-DAG	Yes
Palmitoyl-linoleoyl-glycerol (16:0/18:2) (2)**	0.057	4.9E-05	2.4E10-5	DAG	1,2-DAG	Yes
Oleoyl-linoleoyl-glycerol (18:1/18:2) (1)**	0.065	3.4E-06	1.3E10-6	DAG	1,2-DAG	Yes
Oleoyl-linoleoyl-glycerol (18:1/18:2) (2)**	0.061	1.1E-05	4.9E10-6	DAG	1,2-DAG	Yes
DAG (12:0/18:1, 14:0/16:1, 16:0/14:1) (2)**	0.091	9.0E-07	3.0E10-7	DAG	1,2-DAG	Yes
Oleoyl-arachidonoyl-glycerol (18:1/20:4) (1)**	0.095	3.1E-07	8.0E10-8	DAG	1,2-DAG	Yes
Oleoyl-arachidonoyl-glycerol (18:1/20:4) (2)**	0.087	2.4E-06	9.5E10-7	DAG	1,2-DAG	Yes
Oleoyl-oleoyl-glycerol (18:1/18:1) (2)**	0.087	2.7E-06	7.7E10-7	DAG	1,2-DAG	Yes
1-palmitoleoylglycerol (16:1)	0.082	1.6E-07	4.4E10-8	MAG	1-MAG	Yes
1-oleoylglycerol (18:1)	0.057	5.0E-05	3.2E10-5	MAG	1-MAG	Yes
1-myristoylglycerol (14:0)	0.073	2.0E-07	9.8E10-8	MAG	1-MAG	Yes
**Glycerophospholipids**						
1-stearoyl-2-arachidonoyl-GPI (18:0/20:4)	0.058	3.5E-05	1.2E10-5	GPI	PI	Yes
1-palmitoyl-2-oleoyl-GPE (16:0/18:1)	0.085	1.3E-09	4.0E10-10	GPE	PE	Yes
1-palmitoyl-2-docosahexaenoyl-GPE (16:0/22:6)	0.087	2.1E-08	1.2E10-8	GPE	PE	Yes
1-stearoyl-2-docosahexaenoyl-GPE (18:0/22:6)	0.098	3.1E-10	6.1E10-11	GPE	PE	Yes
1-palmitoyl-2-linoleoyl-GPE (16:0/18:2)	0.063	6.6E-06	7.7E10-6	GPE	PE	Yes
1-stearoyl-2-oleoyl-GPE (18:0/18:1)	0.075	9.0E-08	1.7E10-8	GPE	PE	Yes
1-stearoyl-2-linoleoyl-GPE (18:0/18:2)	0.07	5.0E-07	2.2E10-6	GPE	PE	Yes
1-stearoyl-2-arachidonoyl-GPE (18:0/20:4)	0.064	4.2E-06	1.2E10-6	GPE	PE	Yes
1-oleoyl-2-docosahexaenoyl-GPE (18:1/22:6)	0.082	1.0E-05	6.3E10-6	GPE	PE	Yes
1-oleoyl-2-arachidonoyl-GPE (18:1/20:4)	0.078	2.7E-05	3.8E10-5	GPE	PE	Yes
1-stearoyl-GPE (18:0)	0.077	3.6E-08	3.0E10-8	GPE	Lyso-PEth	Yes
1-palmitoyl-GPE (16:0)	0.068	1.2E-06	2.7E10-6	GPE	Lyso-PEth	Yes
1-myristoyl-2-arachidonoyl-GPC (14:0/20:4)	0.092	3.0E-09	2.9E10-10	GPC	PC	Yes
1-myristoyl-2-palmitoyl-GPC (14:0/16:0)	0.065	2.9E-05	2.1E10-5	GPC	PC	Yes
1-palmitoyl-2-palmitoleoyl-GPC (16:0/16:1)	0.064	5.1E-06	9.8E10-7	GPC	PC	Yes
1-palmitoleoyl-GPC (16:1)	0.065	3.5E-06	1.1E10-6	GPC	Lyso-PCho	Yes
1-(1-enyl-palmitoyl)-2-palmitoleoyl-GPC (P-16:0/16:1)	-0.070	4.8 E-07	1.6E10-7	GPC	PlCho	Yes
1-(1-enyl-palmitoyl)-2-linoleoyl-GPC (P-16:0/18:2)	-0.069	9.7 E-07	1.6E10-7	GPC	PlCho	Yes
1-(1-enyl-palmitoyl)-2-oleoyl-GPC (P-16:0/18:1)	-0.063	5.9E-06	1.1E10-6	GPC	PlCho	Yes
**Sphingolipids**						
lactosyl-N-nervonoyl-sphingosine (d18:1/24:1)	-0.081	1.1E-05	2.7E10-6	GSL	Lactosylceramide	Yes
lactosyl-N-palmitoyl-sphingosine (d18:1/16:0)	-0.061	1.3E-05	2.8E10-6	GSL	Lactosylceramide	Yes

Abbreviations: DAG, Diacylglycerol; GPC, Glycerophosphocoline; GPE, Glycerophosphoethanolamine; GPI, Glycerophosphoinositol; GSL, Glycerosphingolipid; Lyso-PCho, Lysophosphatidylcholine; Lyso-PEth, Lysophosphatidylethanolamine; MAG, Monoacylglycerol; PC, Phosphatidylcholine; PE, Phosphatidylethanolamine; PI, Phosphatidylinositol; PlCho, Plasmalogen-Choline; TAG, Triacylglycerol.

*TAG was measured using an enzymatic method.

Beta and P-values were obtained from linear regression.

P-value*: P value adjusted for batch effect, age and fasting glucose.

**The numbers (1) and (2) in parentheses refer to different stereoisomers of the metabolites.

Abbreviations: DAG, Diacylglycerol; GPC, Glycerophosphocoline; GPE, Glycerophosphoethanolamine; GPI, Glycerophosphoinositol; GSL, Glycerosphingolipid; Lyso-PCho, Lysophosphatidylcholine; Lyso-PEth, Lysophosphatidylethanolamine; MAG, Monoacylglycerol; PC, Phosphatidylcholine; PE, Phosphatidylethanolamine; PI, Phosphatidylinositol; PlCho, Plasmalogen-Choline; TAG, Triacylglycerol.

*TAG was measured using an enzymatic method. **The numbers (1) and (2) in parentheses refer to different stereoisomers of the metabolites. Only metabolites that were associated significantly (*P* < 5.8 × 10^−5^) with *GCKR* rs780094-T are shown. Beta and *P* values were obtained from linear regression, and were adjusted for batch effect, P* values were adjusted for batch effect, age and fasting glucose.

#### Glycerophospholipids

We found 19 novel statistically significant associations of *GCKR* rs780094-T with GPLs after the adjustment for batch effect, age and fasting glucose (16 positive associations, and 3 inverse associations with plasmalogen-cholines).

#### Sphingolipids

Two lactosylceramides, lactosyl-N-nervonoyl-sphingosine (d18:1/24:1) and lactosyl-N-palmitoyl-sphingosine (d18:1/16:0) were significantly and negatively associated with *GCKR* rs780094-T.

#### Palmitic acid

*GCKR* rs780094-T was nominally associated with palmitic acid (*P* = 0.033).

We performed further adjustments for all metabolites significantly associated with *GCKR* rs780094-T (Supplementary Tables S4–S5). First, we adjusted *P* values for bath effect, age, and total triglycerides (Supplementary Tables S4–S5, P*), and finally for bath effect, age, total triglycerides, fasting glucose and for all other metabolites (N = 46) showing significant associations with *GCKR* rs780094-T of (Supplementary Tables S4–S5, P**). Since *GCKR* rs780094-T was significantly associated with total triglycerides and other lipids it was not surprising that the *P* values were substantially less significant or non-significant after adding total triglycerides as an independent variable in the model. Similarly, *P* values further weakened when the adjustment was done for all metabolites significantly associated with *GCKR* rs780094 (Supplementary Tables S4–S5).

### Correlations of lipids with insulin sensitivity, body mass index and ALT

We applied linear regression analysis (adjusted for the bath effect and age) to investigate if the metabolites significantly associated with *GCKR* rs780094-T (Tables 1–2) are also significantly associated with insulin sensitivity, BMI and ALT (Supplementary Tables S6–S8). Amino acids or their downstream metabolites (except for serine and 3-aminoisobutyrate), carbohydrates and other metabolites mentioned in Table 1 (except for 3-hydroxubuturate) were associated with an increase in insulin resistance (a decrease in Matsuda ISI), and a majority of these metabolites were also associated with increases in BMI and ALT (Supplementary Tables S6 and S7). All GLs and GPLs, which were positively associated with *GCKR* rs780094-T (Table 2), were associated with an increase in insulin resistance, BMI and ALT, except for 1-stearoyl-GPE (18:0) and 1-palmitoyl-GPE (16:0) which are lysophoshatidylethanolamines. Plasmalogen-cholines 1-(1-enyl-palmitoyl)-2-palmitoleoyl-GPC (P-16:0/16:1), 1-(1-enyl-pamitoyl)-2-linoleoyl-GPC (P-16:0/18:2), 1-(1-enyl-pamitoyl)-2-oleoyl-GPC (P-16:0/18:1)], and sphingolipids lactosyl-N-nervonoyl-spingosine (d18:1/24:1), lactosyl-N-palmitoyl-spingosine (d18:1/16:0) increased insulin sensitivity and decreased BMI and ALT. We performed linear regression analysis also separately for *GCKR* rs780094-T and the *GCKR* rs780094-CC genotype (Supplementary Tables S8 and S9). Associations remained quite similar in these groups.

## Discussion

We and other investigators have previously shown that *GCKR* rs780094 is associated with several metabolites and diseases^2–7^. In the present study, we performed an extensive metabolomics analysis to obtain a comprehensive view of the associations of *GCKR* rs780094-T with several metabolites belonging to different metabolic pathways. We found novel associations of *GCKR* rs780094-T with amino acids and their downstream metabolites (threonine, indolelactate, N-acetyltryptophan), and especially with lipids.

Previous studies have reported associations of *GCKR* rs780094-T with several amino acids^7^. We additionally found novel negative associations of *GCKR* rs780094-T with serine and threonine, and positive associations with the metabolites indolelactate and N-acetyltryptophan in the tryptophan pathway. Indole, the most abundant metabolite of tryptophan, is produced by bacteria species and modulates the secretion of glucagon-like peptidi-1, and thus insulin secretion^13^. N-acetyltryptophan is also an indicator of the gut microbial metabolism^14^. *GCKR* rs780094-T was inversely associated with 3-aminoisobutyrate. This metabolite is generated by the catabolism of valine, and contributes to exercise-induced protection from metabolic diseases in humans^15^.

We found multiple new positive associations of *GCKR* rs780094-T with GLs. Most of these associations were with 1,2-DAG which can be acetylated to form TAG or redirected to generate phospholipids^10^. TAGs can be hydrolyzed to generate DAGs and FFAs, and DAGs to monoacylglyceride (MAG) and FFAs. The cells can store TAG in lipid droplets which prevents lipotoxicity^16,17^.

We also found several new positive associations of *GCKR* rs780094-T with GPLs which included PEs (N = 9), PCs (N = 3), lysophospatidylethanolamine (N = 2), PI (N = 1), and lysophophatidylcholine (N = 1). Given the fact that the degradation of these GPLs results in FFAs, this results in increased *de novo* lipid synthesis and fat accumulation in the liver. We found negative associations of *GCKR* rs780094-T with three plasmalogen-cholines and two lactosylceramides. Plasmalogens have preventive effects against fat accumulation in the liver since they activate PPARα which increases FA oxidation^18^. Impaired FA oxidation leads to increased FFA levels and synthesis of TAGs in the liver. The negative association of *GCKR* rs780094-T with lactosylceramides (d18:1/24:1) and (d18:1/16:0) suggests that the ceramide pathway may contribute indirectly to fat accumulation in the liver, since downregulation of the ceramide pathway results in an increase in the GL/FFA cycling.

Glycolysis stimulates the formation of acetyl-CoA and synthesis of saturated FAs (Fig. 1), and consequently hepatic TAG accumulation either *via de novo* lipid synthesis or by interrupting β-oxidation^19^. Palmitic acid, a saturated fatty acid, is the first product of *de novo* lipid synthesis, promoting inflammation and endoplasmic reticulum stress^20^. *GCKR* rs780094-T has been previously shown to be associated with high levels of palmitic acid^21^. We also found a similar nominally significant positive association of *GCKR* rs780094-T with palmitic acid.

Ceramides are synthesized from the condensation of serine with palmitic acid and are the precursors for the synthesis of lactosylceramides (Fig. 1). The role of lactosylceramides in the development of insulin resistance has not been well characterized. Lematre *et al*. measured 15 ceramide and sphingomyelin species in fasting baseline samples from a prospective cohort of 2086 American Indians^22^. Their study showed that high levels of lactosylceramide 16 associated with lower fasting insulin, HOMA-IR, and HOMA-B compared to other ceramide species in cross-sectional analyses^22^. This could be explained by the fact that lactosylceramides are ganglioside precursors^23^ which enhance insulin sensitivity^24^. Another study performed in a rodent model showed that lactosylceramide (d18:1/24:1) had a negative correlation with HOMA-IR, although this association was not statistically significant^25^. We found a positive correlation of the lactosylceramides d18:1/24:1 and d18:1/16:0 with insulin sensitivity, and therefore our results agree with previous studies^22,25^ suggesting that lactosylceramides may increase insulin sensitivity. We found low levels of serine and lactosylceramides in the carriers of *GCKR* rs780094-T, which could result in increased level of palmitic acid^26^ and the glycerolipids/FFA cycling^11^, and accumulation of TAGs in the liver.

We found that *GCKR* rs780094-T was inversely associated with gamma-glutamylthreonine which is a dipeptide composed of gamma-glutamate and threonine. A recent study reported that dietary sucrose was positively associated with gamma-glutamylthreonine^27^. We also found a significant positive association of *GCKR* rs780094-T with retinol (vitamin A). Retinol stimulates the secretion of RBP4 protein by hepatocytes^28^, and stimulates *de novo* lipogenesis^29^, thus promoting fat accumulation into the liver.

**Figure 2:**
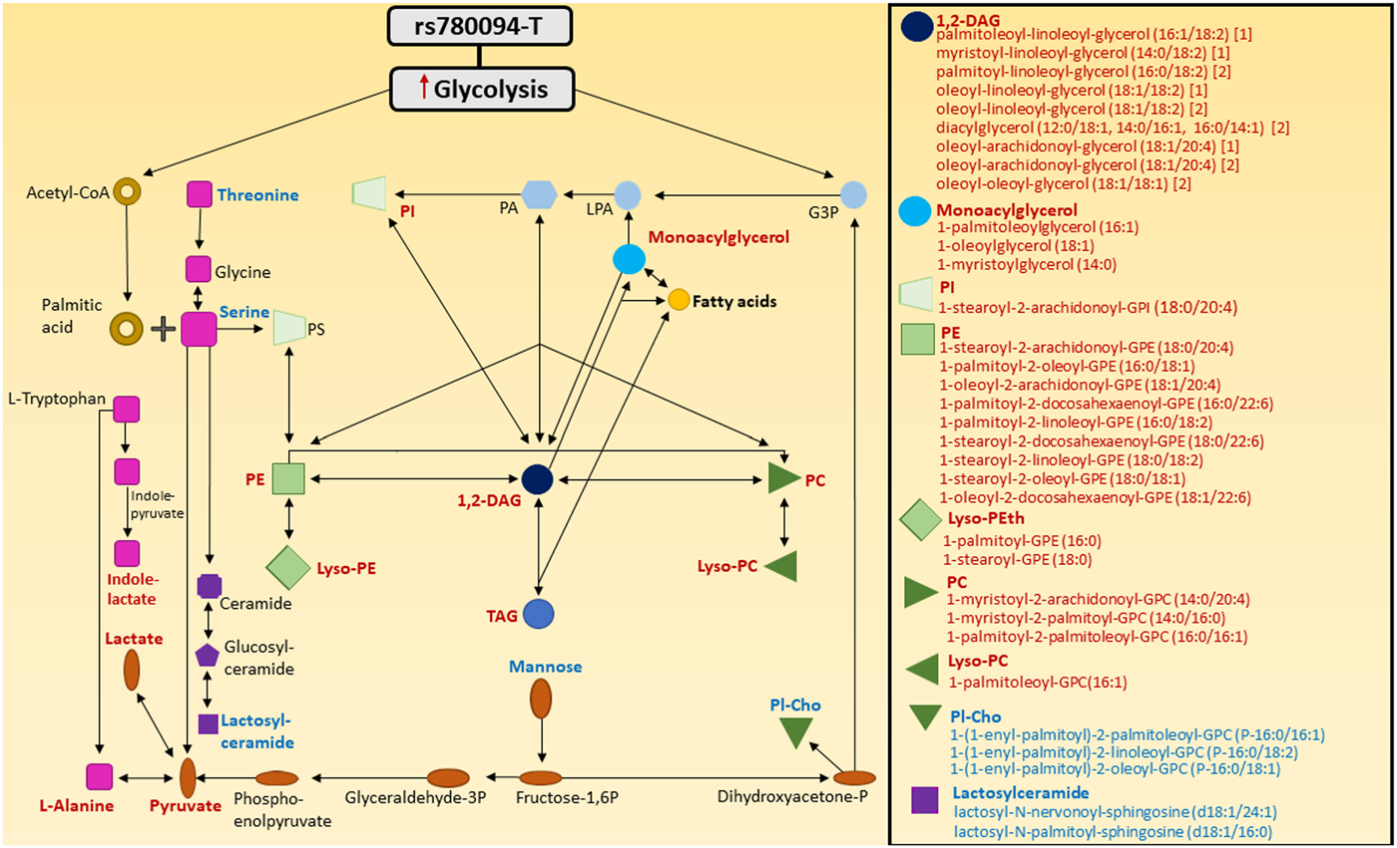
Summary of the associations of rs780094-T of *GCKR* with metabolites in lipid, carbohydrate and amino acid pathways. Increased metabolite levels are marked by red color, decreased metabolite levels by blue color, and other metabolites by black color. A variant rs780094-T of *GCKR* is associated with increased glycolysis and glycogen synthesis, and *de novo* lipid synthesis. Serine is a key metabolite connecting amino acid, lipid and carbohydrate pathways. Low availability of serine and increased palmitic acid increase lipid levels. Serine can be deaminated to pyruvate and further to lactate or alanine. Mannose generates fructose-1,6P which can through multiple reactions generate pyruvate. Dihydroxyacetone-P is the precursor for Pl-Cho and G3P, showing that mannose is a connector between carbohydrate and lipid pathways. Tryptophan can be converted *via* several steps to alanine and also to indole-lactate. Threonine can be metabolized to generate glycine and serine. Acetyl-CoA, Acetyl coenzyme A; DAG, Diacylglycerol; G3P, Glycerol-3-phosphate; GPC, Glycerophosphocholine; GPE, Glycerophosphoethanolamine; GPI, Glycerophosphoinositol; LPA, Lysophosphatidic acid; Lyso-PC, Lysophosphatidylcholine; Lyso-PE, Lysophosphatidylethanolamine; PA, Phosphatidic acid; PC, Phosphatidylcholine; PE, Phosphatidylethanolamine; PI, Phosphatidylinositol; Pl-Cho, Choline plasmalogen; PS, Phosphatidylserine; TAG, Triacylglycerol.

Figure 2 summarizes the associations of *GCKR* rs780094-T with the metabolites belonging to amino acid, carbohydrate and lipid pathways in our study. *GCKR* rs780094-T was associated with the metabolites from all these pathways. Serine is a key metabolite connecting amino acid, lipid and carbohydrate pathway. In our study *GCKR* rs780094-T was inversely associated with serine but positively associated with multiple GLs and GPLs in agreement with the stimulation of *de novo* lipogenesis as previously reported^26^. Serine has also effects on carbohydrate metabolism because it can be deaminated to pyruvate and further to lactate. *GCKR* rs780094-T was inversely associated also with threonine which is expected because this amino acid can be converted to serine.

Our study may have clinical significance for NAFLD/NASH. Variants of the genes that encode proteins in the lipogenesis pathway have been associated with NAFLD and NASH, including a missense variant rs738409 in the patatin like phospholipase 3 (*PNPLA3*)^30^ and rs58542926 in the transmembrane 6 superfamily member 2 (*TM6SF2*) genes^31^. *GCKR* rs780094, which is in linkage disequilibrium with rs126036, is also involved in the NAFLD/NASH pathogenesis, since it activates *de novo* lipogenesis, TAG and cholesterol synthesis^32^. Increased *de novo* lipid synthesis together with impaired β-oxidation leads to fat accumulation in the liver. We found multiple positive associations of *GCKR* rs780094-T with GLs and GLPs, and these lipids were also associated with insulin resistance, obesity and elevation of ALT concentrations. Several of them, e.g. palmitoyl-linoleoyl-glycerol (16:0/18:2), oleoyl-linoleoyl-glycerol (18:1/18:2), oleoyl-oleoyl-glycerol (18:1/18:1), 1-palmitoyl-2-docosahexaenoyl-GPE (16:0/22:6), and 1-stearoyl-2-docosahexaenoyl-GPE (18:0/22:6) have been previously associated in plasma and liver biopsies with NAFLD or NASH^33^. In another study total hepatic lipid content was markedly increased in NAFLD and NASH (*P* < 0.001), driven mainly by increased TAG and DAG^34^. Interestingly, a recent report demonstrated that a rare loss-of-function variant Arg227Ter of *GCKR* was associated with a rapidly progressive form of nonalcoholic steatohepatitis giving further evidence on the role of *GCKR* in liver diseases^35^.

The strength of our study is a large size of our population-based study and detailed metabolite analyses. The limitations of our study are that our study was cross-sectional, and only middle-aged and elderly Finnish men were included in the study. Therefore, we do not know if the results are valid for women, all age groups and other ethnic and racial groups. In conclusion, our study adds novel information about the pleiotropy of the *GCKR* gene, and suggests a role of this gene in the regulation of lipids. These results may add to our understanding how *GCKR* rs780094-T is associated with the risk of liver diseases, especially NAFLD.

## Materials and Methods

### Subjects

The METSIM study comprises 10,197 Finnish men randomly selected from the population register of Kuopio, Eastern Finland, aged from 45 to 73 years, and examined in 2005–2010. The study design has been described previously^12,36^. Our study includes 5,181 participants of the METSIM study without diabetes at baseline (age 57 ± 7 years, body mass index 26.5 ± 3.5 kg/m^2^, mean ± standard deviation). Their fasting plasma glucose was 5.6 ± 0.4 mmol/l and 2-hour glucose 5.9 ± 1.6 mmol/l. This subset of the METSIM study had similar clinical and laboratory characteristics as the entire METSIM population, and therefore it is a representative of the entire METSIM cohort. At baseline, 40% men had normal glucose tolerance in an oral glucose tolerance test (OGTT) (N = 2,092), 46.5% had isolated impaired fasting glucose (IIFG, N = 2,430), 4.1% had isolated impaired glucose tolerance (IIGT, N = 213), and 8.5% had IFG + IGT (N = 446), according to American Diabetes Association criteria^37^. The study was approved by the Ethics Committee of the University of Kuopio and Kuopio University Hospital. All study participants gave written informed consent.

### Methods

All laboratory methods, including metabolomics analysis, were performed in accordance with the relevant guidelines and regulations.

### Clinical and laboratory measurements

Height was measured without shoes to the nearest 0.5 cm. Weight was measured in light clothing with a calibrated digital scale (Seca 877, Hamburg, Germany). Body mass index (BMI) was calculated as weight (kg) divided by height (m) squared. Laboratory studies after 12 h fasting included the following measurements, plasma glucose and insulin, lipids, lipoproteins, and mass spectrometry metabolomics (Metabolon, Durham, NC). An OGTT was performed to evaluate glucose tolerance (75 g of glucose). Clinical and laboratory measurement methods have been previously published^36^. Briefly, plasma glucose was measured by enzymatic hexokinase photometric assay (Konelab Systems Reagents, Thermo Fischer Scientific, Vantaa, Finland). Insulin was determined by immunoassay (ADVIA Centaur Insulin IRI, no 02230141, Siemens Medical Solutions Diagnostics, Tarrytown, NY, USA). Serum alanine aminotransferase (ALT) was measured by an enzymatic photometric test (Konelab Reagent System, Thermo Fisher Scientific, Vantaa, Finland).

### Metabolomics analysis

Metabolites were measured by using Metabolon Inc.’s untargeted Discovery HD4 platform based on Ultrahigh Performance Liquid Chromatography-Tandem Mass Spectroscopy (UPLC-MS/MS) (Metabolon, Morrisville, NC, USA). Samples stored at −80 C prior analysis were prepared using the automated MicroLab STAR® system from Hamilton Company. Several recovery standards were added prior to the first step in the extraction process for quality control (QC) purposes. A pooled matrix sample generated by taking a small volume of each experimental sample served as a technical replicate throughout the data set. Extracted water samples served as process blanks, and QT standards that were carefully chosen not to interfere with the measurement of endogenous compounds were spiked into every analyzed sample, allowed instrument performance monitoring and aided chromatographic alignment. Overall process variability was determined by calculating the median relative standard deviation for all endogenous metabolites present in 100% of the pooled matrix samples. Data normalization step was performed to correct variation resulting from instrument inter-day tuning differences in studies spanning multiple days. Experimental samples were randomized across the platform run with QC samples spaced evenly. Raw data was extracted, peak-identified and QC processed using Metabolon’s hardware and software, and peaks quantified using area-under-the-curve. Compounds were identified by comparison to library entries of purified standards or recurrent unknown entities. Library matches for each compound were checked for each sample and corrected if necessary. Each metabolite was rescaled to set the median equal to 1. After standardization of the metabolite missing values were replaced with the minimum value for each metabolite. The average percent of imputed metabolites among all metabolites was 5%.

The determination of metabolites was performed in three batches. Batch one included 999 samples with 717 metabolites identified, batch two 1,231 samples with 778 metabolites, and batch three 3,000 samples with 843 metabolites identified. All metabolites having >50% missing values were omitted in statistical analyses. A total of 857 unique metabolites were included in current statistical analysis. The sub-classification of the lipids was based on the Human Metabolome Database (http://www.hmdb.ca).

### Calculations

The Matsuda insulin sensitivity index (ISI) was calculated as previously described^36,38^. The selection of Matsuda ISI as a marker of insulin sensitivity was based on our previous validation study^36^.

### Genotyping

We genotyped rs780094 using specific TaqMan assays (ThermoFisher) in a 7500 Real-Time PCR System (Applied Biosystems) as previously described^4^. *GCKR* variants rs780094 and rs1260326 are in high linkage disequilibrium (0.91 in the METSIM study), and therefore the results are shown only for rs780094. The frequency of the minor T allele of rs780094 of the *GCKR* gene (*GCKR* rs780094-T) was 38.4% in our study (N = 5,181).

### Statistical analysis

All statistical analyses were performed using IBM SPSS Statistics 25. We performed association analyses between *GCKR* rs780094-T and metabolites using linear regression analysis adjusted for batch effect, and confounding risk factors. We give the results as standardized beta coefficients and *P* values with the metabolite as a dependent variable. We used Pearson correlation analysis to analyze the inter-correlations of metabolites of interest, and principal component analyses with Varimax rotation to evaluate the number of components formed from metabolites included in statistical analyses. All variables were log-transformed to correct for their skewed distribution. Pearson correlation plot was generated using ggplot2 package based on R. *P* < 5.8 × 10^−5^ was considered as statistically significant given 857 metabolites measured, and *P* < 0.05 as nominally significant.

## Supplementary information

Supplementary Information

## Data Availability

All datasets generated during the current study can be found within the manuscript or the Supplementary Information. Other datasets generated during and/or analysed during the current study are available from the corresponding author on reasonable request.

## References

[1] Flannick J, Florez JC (2016). Type 2 diabetes genetic data sharing to advance complex disease research. Nature Reviews Genetics.

[2] Stančáková A (2011). Effects of 34 risk loci for type 2 diabetes or hyperglycemia on lipoprotein subclasses and their composition in 6,580 nondiabetic Finnish men. Diabetes.

[3] Suhre K (2011). Human metabolic individuality in biomedical and pharmaceutical research. Nature.

[4] Stancáková A (2012). Hyperglycemia and a common variant of GCKR are associated with the levels of eight amino acids in 9,369 Finnish men. Diabetes.

[5] Mahendran Y (2013). Association of ketone body levels with hyperglycemia and type 2 diabetes in 9,398 Finnish men. Diabetes.

[6] Tan HL (2014). Association of glucokinase regulatory gene polymorphisms with risk and severity of non-alcoholic fatty liver disease: an interaction study with adiponutrin gene. J Gastroenterol..

[7] Brouwers MCGJ, Chantal J, Bast A, Stehouwer CDA, Schape NC (2015). Modulation of glucokinase regulatory protein: a double-edged sword?. Trends Mol Med..

[8] Zelent B (2014). Analysis of the co-operative interaction between the allosterically regulated proteins GK and GKRP using tryptophan fluorescence. Biochem. J.

[9] Agius L, Peak M, Newgard CB, Gomez-Foix AM, Guinovart JJ (1996). Evidence for a role of glucose-induced translocation of glucokinase in the control of hepatic glycogen synthesis. J Biol Chem..

[10] Prentki M, Madiraju SR (2008). Glycerolipid metabolism and signaling in health and disease. Endocrine Rev..

[11] Hermansson M, Hokynar K, Somerharju P (2011). Review: Mechanisms of glycerophospholipid homeostasis in mammalian cells. Prog Lipid Res..

[12] Laakso M (2017). The Metabolic Syndrome in Men study: a resource for studies of metabolic and cardiovascular diseases. J Lipid Res..

[13] Chimerel C (2014). Bacterial metabolite indole modulates incretin secretion from intestinal enteroendocrine L cells. Cell Rep..

[14] Pavlova T (2017). Urinary intermediates of tryptophan as indicators of the gut microbial metabolism. Anal Chim Acta..

[15] Roberts LD (2014). β-aminoisobutyric acid induces browning of white fat and hepatic β-oxidation and Is inversely correlated with cardiometabolic risk factors. Cell Metab..

[16] Listenberger LL (2003). Triglyceride accumulation protects against fatty acid-induced lipotoxicity. Proc Natl Acad Sci USA.

[17] Dugail I, Hajduch EA (2007). new look at adipocyte lipid droplets: towards a role in the sensing of triacylglycerol stores?. Cell Mol Life Sci..

[18] Jang JE (2017). Protective role of endogenous plasmalogens against hepatic steatosis and steatohepatitis. Hepatology.

[19] Rui L (2014). Energy metabolism in the liver. Compr Physiol..

[20] Wei Y, Wang D, Topczewski F, Pagliassotti MJ (2006). Saturated fatty acids induce endoplasmic reticulum stress and apoptosis independently of ceramide in liver cells. Am J Physiol Endocrinol Metab..

[21] Wu JHY (2013). Genome-wide association study identifies novel loci associated with concentrations of four plasma phospholipid fatty acids in the de novo lipogenesis pathway: results from the Cohorts for Heart and Aging Research in Genomic Epidemiology (CHARGE) consortium. Circ Cardiovasc Genet..

[22] Lemaitre RN (2018). Circulating sphingolipids, insulin, HOMA-IR, and HOMA-B: The Strong Heart Family Study. Diabetes.

[23] Yu RK, Tsai YT, Ariga T, Yanagisawa M (2011). Structures, biosynthesis, and functions of gangliosides: an overview. J Oleo Sci..

[24] Chavez JA (2014). Ceramides and glucosylceramides are independent antagonists of insulin signaling. J Biol Chem..

[25] Wigger L (2017). Plasma dihydroceramides are diabetes susceptibility biomarker candidates in mice and humans. Cell Rep..

[26] Gao X (2018). Serine availability influences mitochondrial dynamics and function through lipid metabolism. Cell Rep..

[27] Zheng Y, Yu B, Alexander D, Steffen LM, Boerwinkle E (2014). Human metabolome associates with dietary intake habits among African Americans in the atherosclerosis risk in communities study. Am J Epidemiol..

[28] Ronne H (1983). Ligand-dependent regulation of intracellular protein transport: Effect of vitamin A on the secretion of the retinol-binding protein. J Cell Biol..

[29] Xia M (2013). Retinol binding protein 4 stimulates hepatic SREBP-1 and increases lipogenesis through PGC-1beta-dependent pathway. Hepatology.

[30] Santoro N (2010). A common variant in the patatin-like phospholipase 3 gene (PNPLA3) is associated with fatty liver disease in obese children and adolescents. Hepatology.

[31] Macaluso FS, Maida M, Petta S (2015). Genetic background in nonalcoholic fatty liver disease: A comprehensive review. World J Gastroenterol..

[32] Rees MG (2012). Cellular characterization of the GCKR P446L variant associated with type 2 diabetes risk. Diabetologia.

[33] Gorden DL (2015). Biomarkers of NAFLD progression: a lipidomics approach to an epidemic. J Lipid Res..

[34] Puri P (2007). A lipidomic analysis of nonalcoholic fatty liver disease. Hepatology.

[35] Pirola CJ (2018). A rare nonsense mutation in the glucokinase regulator gene is associated with a rapidly progressive clinical form of nonalcoholic steatohepatitis. Hepatol Commun..

[36] Stancáková A (2009). Changes in insulin sensitivity and insulin release in relation to glycemia and glucose tolerance in 6,414 Finnish men. Diabetes.

[37] American Diabetes Association (2013). Diagnosis and classification of diabetes mellitus. Diabetes Care.

[38] Matsuda M, DeFronzo RA (1999). Insulin sensitivity indices obtained from oral glucose tolerance testing: Comparison with the euglycemic insulin clamp. Diabetes Care.

