# Supplementary material for: An intronic variant in the GCKR gene is associated with multiple lipids
Source: Sci Rep. 2019 Jul 15;9:10240. doi: 10.1038/s41598-019-46750-3 (PMC6629684; doi:10.1038/s41598-019-46750-3)
Supplement: Supplementary file 1 — Supplementary Information [file 41598_2019_46750_MOESM1_ESM.pdf]

SUPPLEMENTARY INFORMATION

An intronic variant in the *GCKR* gene is associated with multiple lipids

Lilian Fernandes Silva, M.Sc., Jagadish Vangipurapu, Ph.D., Teemu Kuulasmaa, M.Sc., Markku Laakso, M.D., Ph.D.

Supplementary Figure S1. Heat map showing the correlations between the lipids

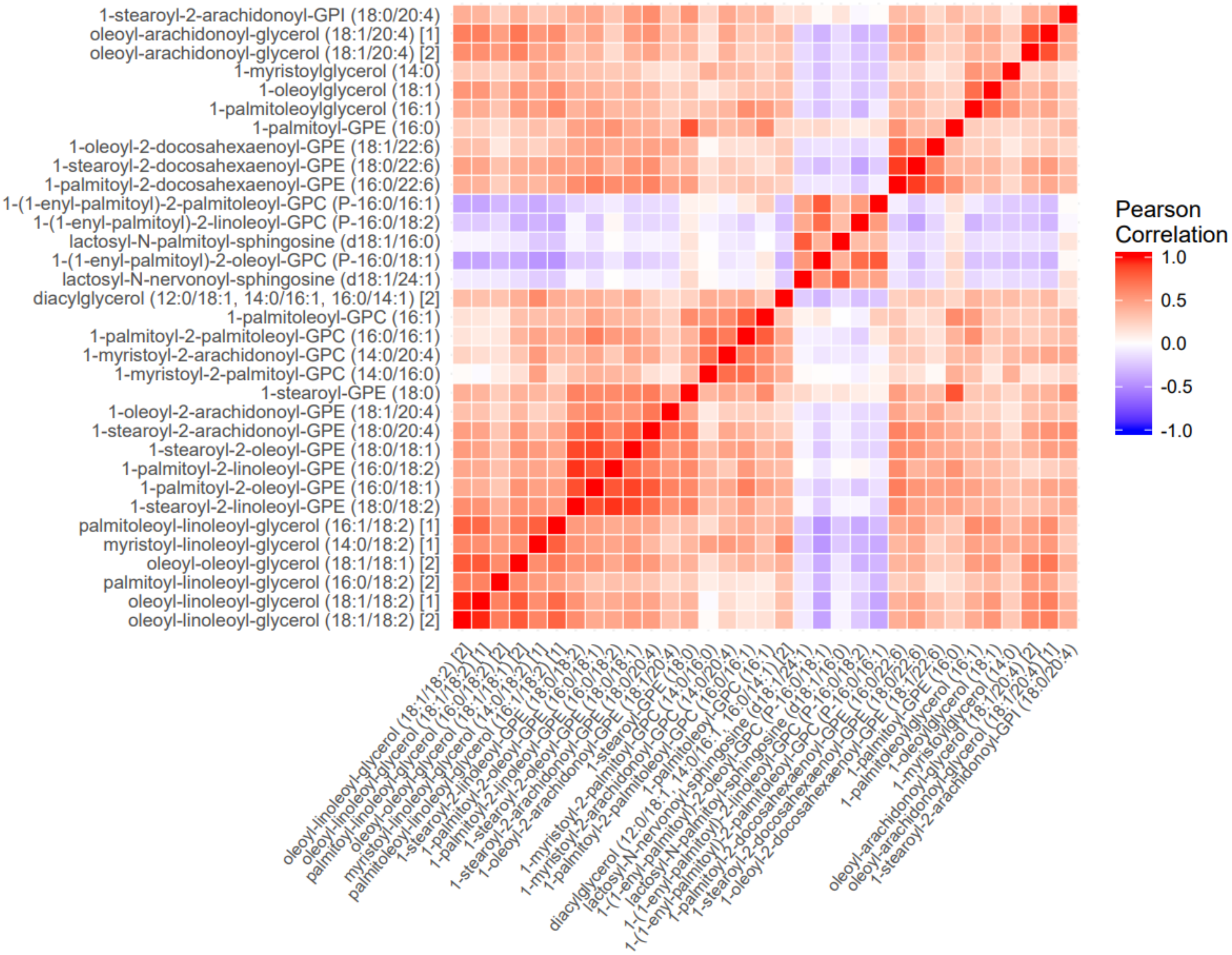

Supplementary Table S1. Principal component analysis of different lipids

	Principal component						
	1	2	3	4	5	6	7
Oleoyl-linoleoyl-glycerol (18:1/18:2) (2)	0,856						
Oleoyl-linoleoyl-glycerol (18:1/18:2) (1)	0,852						
Palmitoyl-linoleoyl-glycerol (16:0/18:2) (2)	0,762						
Oleoyl-oleoyl-glycerol (18:1/18:1) (2)	0,683	0,328					
Myristoyl-linoleoyl-glycerol (14:0/18:2) (1)	0,637		0,534				
Palmitoleoyl-linoleoyl-glycerol (16:1/18:2) (1)	0,629					0,382	
1-stearoyl-2-linoleoyl-GPE (18:0/18:2)	0,377	0,816					
1-palmitoyl-2-oleoyl-GPE (16:0/18:1)		0,800	0,334				
1-palmitoyl-2-linoleoyl-GPE (16:0/18:2)		0,781					
1-stearoyl-2-oleoyl-GPE (18:0/18:1)		0,766					
1-stearoyl-2-arachidonoyl-GPE (18:0/20:4)		0,765					0,384
1-oleoyl-2-arachidonoyl-GPE (18:1/20:4)		0,737					
1-stearoyl-GPE (18:0)		0,521	0,373	0,359	0,320		
1-myristoyl-2-palmitoyl-GPC (14:0/16:0)			0,911				
1-myristoyl-2-arachidonoyl-GPC (14:0/20:4)			0,773				0,038
1-palmitoyl-2-palmitoleoyl-GPC (16:0/16:1)		0,347	0,753			0,368	
1-palmitoleoyl-GPC (16:1)*			0,648			0,392	0,311
Diacylglycerol (12:0/18:1, 14:0/16:1, 16:0/14:1) (2)	0,375		0,538				
Lactosyl-N-nervonoyl-sphingosine (d18:1/24:1)				0,835			
1-(1-enyl-palmitoyl)-2-oleoyl-GPC (P-16:0/18:1)	-0,418			0,784			
Lactosyl-N-palmitoyl-sphingosine (d18:1/16:0)				0,749			
1-(1-enyl-palmitoyl)-2-linoleoyl-GPC (P-16:0/18:2)				0,717			
1-(1-enyl-palmitoyl)-2-palmitoleoyl-GPC (P-16:0/16:1)	-0,472			0,676			
1-palmitoyl-2-docosahexaenoyl-GPE (16:0/22:6)		0,396			0,831		
1-stearoyl-2-docosahexaenoyl-GPE (18:0/22:6)					0,782		
1-oleoyl-2-docosahexaenoyl-GPE (18:1/22:6)		0,349			0,757		
1-palmitoyl-GPE (16:0)		0,306	0,444	0,390	0,493		
1-palmitoleoylglycerol (16:1)						0,800	
1-oleoylglycerol (18:1)	0,392					0,749	
1-myristoylglycerol (14:0)			0,380			0,501	
Oleoyl-arachidonoyl-glycerol (18:1/20:4) (2)	0,530						0,635
Oleoyl-arachidonoyl-glycerol (18:1/20:4) (1)	0,546						0,611
1-stearoyl-2-arachidonoyl-GPI (18:0/20:4)		0,380					0,565
Total variance explained 77.2%	% 38.5	12.6	8.0	6.6	4.3	3.9	3.2

Supplementary Table S2. Nominally significant associations (*P*<0.05) of *GCKR* rs780094-T with metabolites

Metabolite	SE	Beta	*P* value	Metabolite	SE	Beta	*P* value
Asparagine	0,020	-0,055	8,0E-05	1-palmitoyl-2-arachidonoyl-GPI (16:0/20:4)*	0,020	0,048	5,8E-04
(N(1) + N(8))-acetylspermidine	0,027	-0,073	8,0E-05	Tryptophan	0,020	0,048	5,9E-04
1-(1-enyl-palmitoyl)-2-palmitoyl-GPC (P-16:0/16:0)*	0,020	-0,055	9,0E-05	Cysteinylglycine	0,020	0,048	6,0E-04
3-methyl-2-oxovalerate	0,020	0,055	1,0E-04	1-oleoyl-2-linoleoyl-GPE (18:1/18:2)*	0,020	0,048	6,0E-04
1-myristoyl-2-docosahexaenoyl-GPC (14:0/22:6)*	0,041	0,112	1,0E-04	Adenosine	0,041	0,098	6,2E-04
1-palmitoyl-2-arachidonoyl-GPE (16:0/20:4)*	0,020	0,055	1,0E-04	Ribitol	0,020	0,048	6,3E-04
Sphingomyelin (d17:1/16:0, d18:1/15:0, d16:1/17:0)*	0,020	-0,053	1,4E-04	Gamma-glutamylglutamine	0,019	-0,048	6,4E-04
Oleoyl-oleoyl-glycerol (18:1/18:1) [1]*	0,027	0,070	1,6E-04	1-stearoyl-2-docosahexaenoyl-GPC (18:0/22:6)	0,023	0,053	6,7E-04
1-myristoyl-2-linoleoyl-GPC (14:0/18:2)*	0,041	0,108	1,6E-04	3-methoxytyrosine	0,020	-0,048	6,8E-04
N-acetylputrescine	0,020	0,053	1,7E-04	Linoleoyl-linoleoyl-glycerol (18:2/18:2) [1]*	0,027	0,063	7,0E-04
Deoxycholate	0,020	0,053	1,7E-04	Glucuronide of piperine metabolite C17H21NO3 (4)*	0,027	0,063	7,4E-04
Linoleoyl-arachidonoyl-glycerol (18:2/20:4) [1]*	0,027	0,069	1,8E-04	10-undecenoate (11:1n1)	0,020	-0,047	8,7E-04
Glycerophosphoethanolamine	0,020	0,052	1,8E-04	5-oxoproline	0,020	-0,046	0,001
Gamma-tocopherol/beta-tocopherol	0,020	0,052	2,1E-04	1-palmitoyl-GPC (16:0)	0,020	0,046	0,001
Carotene diol (2)	0,027	-0,069	2,1E-04	2-hydroxy-3-methylvalerate	0,020	0,046	0,001
1-oleoyl-GPE (18:1)	0,020	0,052	2,3E-04	Heptenedioate (C7:1-DC)*	0,027	-0,061	0,001
2-keto-3-deoxy-gluconate	0,027	-0,068	2,3E-04	1-palmitoyl-2-adrenoyl-GPC (16:0/22:4)*	0,041	0,094	0,001
Glucuronide of piperine metabolite C17H21NO3 (5)*	0,027	0,068	2,4E-04	1-stearoyl-GPG (18:0)	0,027	0,060	0,001
Glycodeoxycholate	0,020	0,051	2,5E-04	Gamma-glutamylcitrulline*	0,026	-0,060	0,001
1-palmitoyl-2-oleoyl-GPC (16:0/18:1)	0,020	0,051	2,6E-04	Erythronate*	0,020	0,045	0,001
Glucuronide of piperine metabolite C17H21NO3 (3)*	0,027	0,068	2,6E-04	Myristoyl-linoleoyl-glycerol (14:0/18:2) [2]*	0,027	0,060	0,001
Palmitoyl sphingomyelin (d18:1/16:0)	0,020	-0,051	2,7E-04	2-stearoyl-GPE (18:0)*	0,020	0,045	0,001
Taurochenodeoxycholate	0,020	-0,051	2,8E-04	1-(1-enyl-palmitoyl)-2-arachidonoyl-GPC (P-16:0/20:4)*	0,020	-0,045	0,001
Stearidonate (18:4n3)	0,020	0,051	3,0E-04	Octadecadienedioate (C18:2-DC)*	0,027	-0,059	0,001
2-hydroxybutyrate/2-hydroxyisobutyrate	0,020	0,051	3,0E-04	Butyrylcarnitine (C4)	0,020	0,044	0,002
Glutamine	0,019	-0,050	3,3E-04	1-palmitoyl-2-docosahexaenoyl-GPC (16:0/22:6)	0,023	0,049	0,002
Linoleoyl-arachidonoyl-glycerol (18:2/20:4) [2]*	0,027	0,066	3,7E-04	1-linolenoyl-GPC (18:3)*	0,020	0,043	0,002
Palmitoyl-linoleoyl-glycerol (16:0/18:2) [1]*	0,020	0,050	3,8E-04	2-palmitoyl-GPC (16:0)*	0,020	0,043	0,002
Docosapentaenoate (n3 DPA; 22:5n3)	0,020	0,050	3,9E-04	Pipecolate	0,020	-0,043	0,002
4-guanidinobutanoate	0,020	-0,050	3,9E-04	Propionylcarnitine (C3)	0,020	0,043	0,002
Pantothenate	0,020	0,050	3,9E-04	1-stearoyl-2-docosapentaenoyl-GPC (18:0/22:5n3)*	0,041	0,087	0,002
1-palmitoleoyl-2-linolenoyl-GPC (16:1/18:3)*	0,023	0,056	4,3E-04	1-myristoyl-GPC (14:0)	0,041	0,087	0,002
Octadecenedioate (C18:1-DC)*	0,027	-0,064	5,1E-04	4-methyl-2-oxopentanoate	0,020	0,042	0,002

Erythritol	0,020	0,042	0,002	N-acetylglycine	0,020	-0,036	0,010
Isoleucine	0,020	0,042	0,003	1-arachidonoyl-GPI (20:4)*	0,020	0,036	0,010
Hypotaurine	0,020	0,042	0,003	Behenoyl dihydrosphingomyelin (d18:0/22:0)*	0,027	0,047	0,011
3b-hydroxy-5-cholenoic acid	0,022	-0,046	0,003	Uracil	0,020	0,036	0,011
1-stearoyl-2-oleoyl-GPC (18:0/18:1)	0,020	0,041	0,003	Methionine sulfoxide	0,020	-0,036	0,011
1-(1-enyl-stearoyl)-2-linoleoyl-GPE (P-18:0/18:2)*	0,020	-0,041	0,003	Gamma-glutamylglycine	0,020	-0,036	0,011
Sphingomyelin (d18:2/24:2)*	0,027	-0,054	0,003	2-hydroxydecanoate	0,020	-0,035	0,011
Palmitoyl dihydrosphingomyelin (d18:0/16:0)*	0,020	-0,041	0,003	Sphingomyelin (d18:0/20:0, d16:0/22:0)*	0,027	0,047	0,011
4-methylbenzenesulfonate	0,041	0,084	0,003	1-(1-enyl-oleoyl)-GPC (P-18:1)*	0,041	-0,072	0,012
Carotene diol (1)	0,027	-0,054	0,003	Palmitoylcarnitine (C16)	0,020	0,035	0,012
1-palmitoyl-GPG (16:0)*	0,022	0,045	0,004	Urate	0,020	0,035	0,012
Alpha-ketobutyrate	0,020	0,040	0,004	Quinate	0,020	-0,035	0,012
1-linoleoyl-GPE (18:2)*	0,020	0,039	0,005	Myristate (14:0)	0,020	0,035	0,012
3-methylglutarylcarnitine (2)	0,020	0,039	0,005	3-hydroxyhexanoate	0,020	-0,035	0,013
Eicosanodioate (C20-DC)	0,020	-0,039	0,005	Sphinganine-1-phosphate	0,023	0,040	0,013
Leucine	0,020	0,039	0,006	Sphingomyelin (d18:2/16:0, d18:1/16:1)*	0,020	-0,035	0,013
Citrulline	0,020	-0,038	0,006	Docosahexaenoate (DHA; 22:6n3)	0,020	0,034	0,014
Dihomo-linolenate (20:3n3 or n6)	0,020	0,038	0,007	Dihomo-linolenoylcarnitine (C20:3n3 or 6)*	0,027	0,046	0,014
Alpha-hydroxyisovalerate	0,020	0,038	0,007	Tyrosine	0,020	-0,034	0,014
Glycosyl-N-palmitoyl-sphingosine (d18:1/16:0)	0,020	-0,038	0,007	Dimethylglycine	0,020	-0,034	0,015
N-acetylneuraminate	0,020	0,038	0,007	Arachidonoylcarnitine (C20:4)	0,027	0,045	0,016
1-dihomo-linolenylglycerol (20:3)	0,020	0,037	0,007	Gamma-carboxyglutamate	0,020	0,034	0,016
1-(1-enyl-palmitoyl)-2-linoleoyl-GPE (P-16:0/18:2)*	0,020	-0,037	0,007	Androstenediol (3beta,17beta) monosulfate (2)	0,020	0,033	0,017
1-linolenoylglycerol (18:3)	0,023	0,043	0,007	1-eicosapentaenoyl-GPE (20:5)*	0,041	0,068	0,017
1-stearoyl-2-docosapentaenoyl-GPC (18:0/22:5n6)*	0,041	0,076	0,008	Trigonelline (N'-methylnicotinate)	0,020	-0,033	0,018
N-acetyl-isoputreanine*	0,027	-0,049	0,008	Gamma-glutamyltryptophan	0,022	0,037	0,018
1-margaroyl-2-oleoyl-GPC (17:0/18:1)*	0,041	0,076	0,008	Eicosapentaenoate (EPA; 20:5n3)	0,020	0,033	0,018
Choline phosphate	0,020	0,037	0,008	Histidine	0,020	-0,033	0,018
Arginine	0,020	-0,037	0,008	1-(1-enyl-stearoyl)-2-arachidonoyl-GPC (P-18:0/20:4)	0,041	-0,067	0,019
2-myristoyl-GPC* (14:0)*	0,041	0,075	0,008	6-oxopiperidine-2-carboxylic acid	0,031	0,050	0,019
Cyclo(leu-pro)	0,041	0,075	0,008	1-stearoyl-GPI (18:0)	0,020	0,033	0,019
Sphingomyelin (d18:2/18:1)*	0,027	-0,048	0,009	Flavin adenine dinucleotide (FAD)	0,027	0,043	0,019
Nisinate (24:6n3)	0,027	0,048	0,009	N-acetylmethionine	0,020	-0,033	0,019
1-palmitoyl-2-dihomo-linolenoyl-GPC (16:0/20:3n3 or 6)*	0,023	0,040	0,009	Cholate	0,020	-0,033	0,019
Taurolithocholate 3-sulfate	0,020	-0,036	0,010	1-(1-enyl-stearoyl)-2-linoleoyl-GPC (P-18:0/18:2)*	0,041	-0,067	0,020
Tigloylglycine	0,031	-0,055	0,010	Octadecanedioate (C18-DC)	0,020	-0,032	0,020

3-methyladipate	0,020	-0,032	0,021	Glucose	0,020	-0,032	0,021
1-arachidonoyl-GPE (20:4n6)*	0,020	0,032	0,021	Phosphatidylcholine (15:0/18:1, 17:0/16:1)*	0,041	0,061	0,034
Pyroglutamine*	0,020	-0,032	0,022	1-palmitoyl-2-dihomo-linolenoyl-GPE (16:0/20:3)*	0,041	0,060	0,035
Kynurenate	0,020	-0,032	0,022	1-stearoyl-GPC (18:0)	0,020	0,029	0,036
Ornithine	0,020	-0,032	0,022	1-margaroyl-GPE (17:0)*	0,041	0,060	0,037
3-(3-hydroxyphenyl)propionate sulfate	0,020	-0,032	0,022	Linoleoyl-docosahexaenoyl-glycerol (18:2/22:6) [2]*	0,027	0,039	0,037
Taurine	0,020	0,032	0,022	N6-carboxymethyllysine	0,031	0,044	0,038
Homoarginine	0,020	-0,032	0,023	Phosphatidylcholine (18:0/20:5, 16:0/22:5n6)*	0,041	0,059	0,038
N-methylproline	0,020	0,032	0,023	Thymol sulfate	0,020	-0,029	0,039
Piperine	0,020	0,032	0,023	N6,N6,N6-trimethyllysine	0,020	-0,029	0,039
Beta-alanine	0,020	0,032	0,024	1-oleoyl-GPG (18:1)*	0,027	0,038	0,040
Dimethylarginine (SDMA + ADMA)	0,020	-0,031	0,025	Gamma-glutamylmethionine	0,020	-0,029	0,040
5alpha-androstan-3alpha,17beta-diol monosulfate (2)	0,020	0,031	0,025	Phosphoethanolamine	0,020	0,029	0,040
1-oleoyl-2-docosapentaenoyl-GPC (18:1/22:5n6)*	0,041	0,064	0,025	3-hydroxyadipate*	0,027	-0,038	0,041
Palmitoloelycholine	0,023	0,035	0,026	Malonylcarnitine	0,020	0,029	0,041
Stearoylcarnitine (C18)	0,020	0,031	0,027	Stearate (18:0)	0,020	0,029	0,041
3-phenylpropionate (hydrocinnamate)	0,020	-0,031	0,027	Imidazole propionate	0,020	-0,028	0,042
Margarate (17:0)	0,020	0,031	0,027	S-methylcysteine sulfoxide	0,027	-0,038	0,042
1-methylxanthine	0,020	-0,031	0,027	N-acetyl-beta-alanine	0,020	0,028	0,043
Cysteine	0,020	0,031	0,028	1-linoleoylglycerol (18:2)	0,020	0,028	0,044
Glycochenodeoxycholate sulfate	0,020	-0,031	0,028	2-hydroxynervonate*	0,027	-0,037	0,045
N4-acetylcytidine	0,027	0,041	0,028	1-palmitoleoyl-GPE (16:1)*	0,041	0,058	0,045
Docosapentaenoylcarnitine (C22:5n3)*	0,027	0,040	0,030	Hexanoylglutamine	0,027	-0,037	0,045
Octadecenedioylcarnitine (C18:1-DC)*	0,027	-0,040	0,031	Hexadecadienoate (16:2n6)	0,027	0,037	0,045
Adenosine 5'-monophosphate (AMP)	0,020	0,030	0,032	Caprylate (8:0)	0,031	0,042	0,046
1,2-dilinoleoyl-GPC (18:2/18:2)	0,020	-0,030	0,032	maltotriose	0,020	0,028	0,046
S-adenosylhomocysteine (SAH)	0,022	0,033	0,032	1-(1-enyl-stearoyl)-2-oleoyl-GPC (P-18:0/18:1)	0,041	-0,057	0,048
Palmitate (16:0)	0,020	0,030	0,033	1-docosahexaenoylglycerol (22:6)	0,020	0,028	0,049
Linolenate [alpha or gamma; (18:3n3 or 6)]	0,020	0,030	0,034	3-hydroxylaurate	0,020	0,028	0,049
1-linoleoyl-GPA (18:2)*	0,031	-0,045	0,034	1-oleoyl-2-docosahexaenoyl-GPC (18:1/22:6)*	0,023	0,031	0,049
Adenosine 3',5'-cyclic monophosphate (cAMP)	0,041	0,061	0,034				

Abbreviations: GPC, Glycerophosphocoline; GPE, Glycerophosphoethanolamine; GPG, Glycerophosphoglycerol; GPI, Glycerophosphoinositol; Leu-Pro, Leucine-Proline; P, Plasmalogen.

Beta and *P* values were obtained from linear regression.

P value: adjusted for batch only.

Supplementary Table S3. Non-significant associations (*P*≥0.05) of rs780094 *GCKR*-T with metabolites

**Metabolite**		
eugenol sulfate	N-palmitoyl-heptadecasphingosine (d17:1/16:0)*	phosphatidylcholine (16:0/22:5n3, 18:1/20:4)*
stachydrine	4-acetamidobutanoate	dimethyl sulfone
2-hydroxypalmitate	hexanoylcarnitine (C6)	phenylalanine
hexadecenedioate (C16:1-DC)*	1-(1-enyl-stearoyl)-2-oleoyl-GPE (P-18:0/18:1)	linoleoyl-docosahexaenoyl-glycerol (18:2/22:6) [1]*
uridine	4-hydroxyhippurate	beta-citrylglutamate
2-oleoylglycerol (18:1)	alpha-tocopherol	S-1-pyrroline-5-carboxylate
3beta,7alpha-dihydroxy-5-cholestenoate	2-methylbutyrylcarnitine (C5)	phenol sulfate
sphingomyelin (d18:1/17:0, d17:1/18:0, d19:1/16:0)	phenylacetylglutamine	indoleacetylglutamine
7-alpha-hydroxy-3-oxo-4-cholestenoate (7-Hoca)	beta-hydroxyisovaleroylcarnitine	1-docosahexaenoyl-GPE (22:6)*
4-hydroxyphenylacetylglutamine	N2-methylguanosine	carboxyethyl-GABA
1-dihomo-linoleoylglycerol (20:2)	1-palmitoylglycerol (16:0)	1-stearoyl-2-dihomo-linolenoyl-GPC (18:0/20:3n3 or 6)*
1-(1-enyl-stearoyl)-2-arachidonoyl-GPE (P-18:0/20:4)*	ethyl paraben sulfate	1-dihomo-linolenoyl-GPE (20:3n3 or 6)*
5alpha-androstan-3beta,17beta-diol monosulfate (2)	acisoga	laurate (12:0)
gamma-glutamylphenylalanine	3-hydroxystachydrine*	glycochenodeoxycholate glucuronide (1)
N6-succinyladenosine	N-acetylleucine	cerotoylcarnitine (C26)*
3-hydroxypyridine sulfate	1,2-dilinolenoyl-GPC (18:3/18:3)*	sphingomyelin (d18:1/22:2, d18:2/22:1, d16:1/24:2)*
sphingomyelin (d18:1/25:0, d19:0/24:1, d20:1/23:0, d19:1/24:0)*	2-hydroxyoctanoate	sphingadienine
glycine conjugate of C10H12O2*	succinylcarnitine (C4-DC)	10-heptadecenoate (17:1n7)
gamma-CEHC	lactosyl-N-behenoyl-sphingosine (d18:1/22:0)*	5alpha-androstan-3beta,17beta-diol disulfate
1-(1-enyl-stearoyl)-GPC (P-18:0) *	3-methyl-2-oxobutyrate	2-hydroxystearate
1-palmitoyl-2-oleoyl-GPI (16:0/18:1)*	glycochenodeoxycholate	N-oleoylserine
L-urobilin	5-acetylamino-6-amino-3-methyluracil	suberate (C8-DC)
1-(1-enyl-palmitoyl)-GPE (P-16:0)*	sulfate of piperine metabolite C16H19NO3 (3)*	1-oleoyl-2-dihomo-linolenoyl-GPC (18:1/20:3)*
lignoceroyl sphingomyelin (d18:1/24:0)	3-(4-hydroxyphenyl)lactate	5alpha-androstan-3alpha,17beta-diol monosulfate (1)
1-methylurate	cinnamoylglycine	N-acetyltyrosine
17-methylstearate	isovalerylcarnitine (C5)	docosapentaenoate (n6 DPA; 22:5n6)
2-aminooctanoate	mannitol/sorbitol	4-hydroxyphenylacetate
octadecanedioylcarnitine (C18-DC)*	androsterone glucuronide	gamma-glutamylhistidine

N6-acetyllysine	3-hydroxyoctanoate	5,6-dihydrothymine
maltose	1-palmityl-2-oleoyl-GPC (O-16:0/18:1)*	glutamate
nicotinamide	docosahexaenoylcholine	betaine
N-acetyl-1-methylhistidine*	2-hydroxyhippurate (salicylurate)	methylsuccinoylcarnitine (1)
5alpha-androstan-3beta,17beta-diol monosulfate (1)	1-palmitoyl-2-eicosapentaenoyl-GPC (16:0/20:5)*	N-palmitoyl-sphingadienine (d18:2/16:0)*
N-formylphenylalanine	serotonin	N-acetylserine
succinimide	phenyllactate (PLA)	N-stearoyl-sphingosine (d18:1/18:0)*
3-(3-hydroxyphenyl)propionate	caprate (10:0)	2'-O-methyluridine
ectoine	1-linoleoyl-GPG (18:2)*	1-(1-enyl-palmitoyl)-2-myristoyl-GPC (P-16:0/14:0)*
3-hydroxyisobutyrate	5,6-dihydrouridine	homovanillate (HVA)
N-acetylphenylalanine	5alpha-pregnan-3beta,20alpha-diol monosulfate (2)	N-palmitoylserine
1-oleoyl-GPC (18:1)	cortisol	sphingomyelin (d18:0/18:0, d19:0/17:0)*
ribonate	pristanate	picolinate
hippurate	sulfate*	1-oleoyl-GPI (18:1)*
N-formylmethionine	glycodeoxycholate sulfate	malate
beta-cryptoxanthin	1-stearoyl-2-oleoyl-GPI (18:0/18:1)*	13-HODE + 9-HODE
hexadecanedioate (C16-DC)	prolylglycine	5-methyluridine (ribothymidine)
1-methylnicotinamide	valine	dodecanedioate (C12-DC)
N-methylhydroxyproline**	trans-urocanate	methyl-4-hydroxybenzoate sulfate
glycosyl ceramide (d18:2/24:1, d18:1/24:2)*	3-hydroxyhippurate	S-methylcysteine
sphingomyelin (d18:2/24:1, d18:1/24:2)*	pimeloylcarnitine/3-methyladipoylcarnitine	2-docosahexaenoyl-GPE (22:6)*
1-(1-enyl-palmitoyl)-2-oleoyl-GPE (P-16:0/18:1)*	cysteine-glutathione disulfide	ADP
1-margaroyl-2-docosahexaenoyl-GPC (17:0/22:6)*	gamma-glutamyltyrosine	orotidine
methionine	5alpha-androstan-3alpha,17beta-diol disulfate	salicylate
7-methylxanthine	glycine	phenylalanyltryptophan
1-eicosenoyl-GPC (20:1)*	phosphatidylcholine (14:0/14:0, 16:0/12:0)	palmitoleate (16:1n7)
creatine	indoleacetate	1-arachidoyl-GPC (20:0)
1-arachidonylglycerol (20:4)	ximenoylcarnitine (C26:1)*	dihomo-linolenoyl-choline
1-palmitoyl-2-linoleoyl-GPI (16:0/18:2)	4-hydroxychlorothalonil	arachidonate (20:4n6)
1-palmitoyl-2-alpha-linolenoyl-GPC (16:0/18:3n3)*	alpha-ketoglutarate	cysteine s-sulfate

2-palmitoleoylglycerol (16:1)*	1-pentadecanoyl-GPC (15:0)*	gulonic acid*
linoleoyl ethanolamide	1-eicosapentaenoyl-GPC (20:5)*	xylose
sulfate of piperine metabolite C16H19NO3 (2)*	1-(1-enyl-palmitoyl)-GPC (P-16:0)*	chenodeoxycholate
9-hydroxystearate	sphingosine 1-phosphate	N-acetylisoleucine
isovalerylglycine	ceramide (d16:1/24:1, d18:1/22:1)*	1-stearoyl-2-meadoyl-GPC (18:0/20:3n9)*
alpha-ketoglutaramate*	indoleacetylcarnitine*	xanthurenate
catechol sulfate	4-hydroxy-2-oxoglutaric acid	N-stearoylserine*
5-dodecenoylcarnitine (C12:1)	N-palmitoylglycine	1-linoleoyl-2-linolenoyl-GPC (18:2/18:3)*
EDTA	propionylglycine	1-(1-enyl-oleoyl)-GPE (P-18:1)*
homocitrulline	1-docosapentaenoyl-GPC* (22:5n3)*	citrate
tartronate (hydroxymalonate)	isobutyrylcarnitine (C4)	sphingomyelin (d18:2/14:0, d18:1/14:1)*
linoleate (18:2n6)	3-hydroxy-2-ethylpropionate	stearoylcholine*
alpha-hydroxyisocaproate	2-arachidonoylglycerol (20:4)	valerate (5:0)
1-stearoyl-2-oleoyl-GPS (18:0/18:1)	1,2-dipalmitoyl-GPC (16:0/16:0)	o-cresol sulfate
lanthionine	pregnanediol-3-glucuronide	1,3-dimethylurate
tricosanoyl sphingomyelin (d18:1/23:0)*	4-methylguaiacol sulfate	3,4-methyleneheptanoate
pregnenediol disulfate (C21H34O8S2)*	nonadecanoate (19:0)	1-docosapentaenoyl-GPC* (22:5n6)*
1-linoleoyl-GPC (18:2)	spermidine	5-HEPE
pyroglutamylglutamine	2-aminoadipate	2-stearoyl-GPI (18:0)*
arabonate/xylonate	N2,N5-diacetylornithine	1-pentadecanoyl-2-arachidonoyl-GPC (15:0/20:4)*
oleoylcholine	theophylline	2-linoleoyl-GPC* (18:2)*
sphingomyelin (d18:1/20:2, d18:2/20:1, d16:1/22:2)*	isocitric lactone	maleate
thyroxine	allantoin	oleoyl ethanolamide
1-oleoyl-2-linoleoyl-GPC (18:1/18:2)*	1-palmitoyl-GPI (16:0)	N-acetylglucosamine/N-acetylgalactosamine
gamma-glutamyl-epsilon-lysine	glycosyl ceramide (d16:1/24:1, d18:1/22:1)*	tartarate
N-acetylaspartate (NAA)	3-hydroxymyristate	1-stearoyl-2-arachidonoyl-GPS (18:0/20:4)
heneicosapentaenoate (21:5n3)	sarcosine	16a-hydroxy DHEA 3-sulfate
adipoylcarnitine (C6-DC)	1-palmitoyl-2-stearoyl-GPC (16:0/18:0)	eicosenoylcarnitine (C20:1)*
1-eicosatrienoyl-GPC* (20:3)*	isoleucylglycine	paraxanthine
thioproline	N-stearoyl-sphingadienine (d18:2/18:0)*	methionine sulfone

1-(1-enyl-stearoyl)-GPE (P-18:0)*	myristoylcarnitine (C14)	androstenediol (3alpha, 17alpha) monosulfate (2)
1-pentadecanoyl-2-linoleoyl-GPC (15:0/18:2)*	N-acetylthreonine	5-dodecenoate (12:1n7)
myristoleate (14:1n5)	phosphate	dihomo-linoleoylcarnitine (C20:2)*
5-hydroxyindoleacetate	1-(1-enyl-stearoyl)-2-docosahexaenoyl-GPC (P-18:0/22:6)*	4-cholesten-3-one
N-acetylalanine	1-palmitoleoyl-2-oleoyl-glycerol (16:1/18:1)*	p-cresol-glucuronide*
2-palmitoyl-GPE* (16:0)*	argininate*	kynurenine
sphingomyelin (d18:1/20:0, d16:1/22:0)*	perfluorooctanoate (PFOA)*	3beta-hydroxy-5-cholestenoate
3-carboxy-4-methyl-5-propyl-2-furanpropanoate (CMPF)	1-linoleoyl-2-arachidonoyl-GPC (18:2/20:4n6)*	2-acetamidophenol sulfate
oleate/vaccenate (18:1)	androsterone sulfate	glycerate
1-stearyl-GPC (O-18:0)*	dodecenedioate (C12:1-DC)*	7-methylguanine
isoleucylalanine	indolepropionate	dihomo-linoleate (20:2n6)
dopamine 3-O-sulfate	1-margaroyl-2-linoleoyl-GPC (17:0/18:2)*	5alpha-pregnan-3beta,20alpha-diol disulfate
docosadioate (C22-DC)	N-linoleoyltaurine*	1-heptadecanoyl-GPC (17:0)
cortisone	butyrylglycine (C4)	nicotinamide riboside
3-hydroxy-3-methylglutarate	androstenediol (3beta,17beta) monosulfate (1)	1-(1-enyl-palmitoyl)-2-docosahexaenoyl-GPE (P-16:0/22:6)*
1-ribosyl-imidazoleacetate*	lysine	pregnenediol sulfate (C21H34O5S)*
sphinganine	2-methylbutyrylglycine (C5)	arachidate (20:0)
2-docosahexaenoyl-GPC* (22:6)*	ribulonate/xylulonate*	1-(1-enyl-palmitoyl)-2-arachidonoyl-GPE (P-16:0/20:4)*
azelate (C9-DC)	guanidinoacetate	glycocholate
glycerophosphorylcholine (GPC)	isovalerate (i5:0)	adenine
1,2,3-benzenetriol sulfate (2)	prolylserine	biliverdin
lignoceroylcarnitine (C24)*	orotate	pentadecanoate (15:0)
sphingomyelin (d18:2/21:0, d16:2/23:0)*	glycolithocholate sulfate*	dehydroisoandrosterone sulfate (DHEA-S)
2-methylcitrate/homocitrate	phenol glucuronide	1-nonadecanoyl-GPC (19:0)
N-trimethyl 5-aminovalerate	N-acetylglucosaminylasparagine	1-methylhistidine
isobutyrylglycine	1-eicosadienoyl-GPC* (20:2)*	N-acetyltaurine
2-hydroxyphenylacetate	4-methoxyphenol sulfate	caffeine
aconitate [cis or trans]	aspartate	N-acetylhistidine
hydroquinone sulfate	behenoylcarnitine (C22)*	10-nonadecenoate (19:1n9)

sphingomyelin (d18:2/23:0, d18:1/23:1, d17:1/24:1)*	1,2-distearoyl-GPC (18:0/18:0)	theobromine
fructose	cystathionine	methyl indole-3-acetate
1-stearoyl-GPS (18:0)*	galactonate	3-methoxycatechol sulfate (1)
gamma-glutamyl-alpha-lysine	hydroxy-CMPF*	sphingomyelin (d18:1/19:0, d19:1/18:0)*
N6-carbamoylthreonyladenosine	1-stearoyl-2-arachidonoyl-GPC (18:0/20:4)	1-docosahexaenoyl-GPC* (22:6)*
creatinine	5-acetylamino-6-formylamino-3-methyluracil	N-delta-acetylornithine
2,3-dihydroxy-2-methylbutyrate	cystine	octanoylcarnitine (C8)
indolin-2-one	4-vinylphenol sulfate	inosine 5'-monophosphate (IMP)
N-stearoyltaurine	1,5-anhydroglucitol (1,5-AG)	N6-methyladenosine
3-phosphoglycerate	docosahexaenoylcarnitine (C22:6)*	proline
3,7-dimethylurate	5-hydroxylysine	1-palmitoleoyl-2-linoleoyl-GPC (16:1/18:2)*
perfluorooctanesulfonic acid (PFOS)	1-O-hexadecyl-GPC (C16)	1-stearoyl-2-linoleoyl-GPC (18:0/18:2)*
2-palmitoylglycerol (16:0)	N-acetylkynurenine (2)	gamma-glutamyl-2-aminobutyrate
N-acetylvaline	15-methylpalmitate (i17:0)	sebacate (C10-DC)
1,3,7-trimethylurate	succinate	2-aminobutyrate
eicosapentaenoylcholine	1-(1-enyl-palmitoyl)-2-docosahexaenoyl-GPC (P-16:0/22:6)*	choline
benzoate	4-oxo-retinoic acid	1-linoleoyl-2-docosapentaenyol-GPC (18:2/22:5n3)*
carotene diol (3)	2-arachidonoyl-GPC* (20:4)*	glycerol
tryptophan betaine	glycine conjugate of C10H14O2 (1)*	dodecadienoate (12:2)*
sphingomyelin (d18:1/18:1, d18:2/18:0)	sphingomyelin (d17:1/14:0, d16:1/15:0)*	androstenediol (3beta,17beta) disulfate (1)
sphingomyelin (d18:2/23:1)*	N1-methylinosine	1-lignoceroyl-GPC (24:0)
ceramide (d18:2/24:1, d18:1/24:2)*	N-acetylcarnosine	1-pentadecanoyl-2-docosahexaenoyl-GPC (15:0/22:6)*
sulfate of piperine metabolite C18H21NO3 (3)*	sulfate of piperine metabolite C18H21NO3 (1)*	1-palmitoyl-2-arachidonoyl-GPC (16:0/20:4n6)
1-meadoyl-GPC (20:3n9)*	3-hydroxybutyrylcarnitine (2)	threonate
deoxycarnitine	4-ethylphenylsulfate	4-hydroxycoumarin
1-stearoyl-2-linoleoyl-GPI (18:0/18:2)	suberoylcarnitine (C8-DC)	formiminoglutamate
1-oleoyl-2-eicosapentaenoyl-GPC (18:1/20:5)*	leucylleucine	behenoyl sphingomyelin (d18:1/22:0)*
guanidinosuccinate	docosadienoate (22:2n6)	pregnenolone sulfate
palmitoylcholine	erucate (22:1n9)	sphingomyelin (d18:1/20:1, d18:2/20:0)*

N-methylpipecolate	pyroglutamylvaline	3-methylxanthine
ursodeoxycholate	cis-4-decenoylcarnitine (C10:1)	adrenate (22:4n6)
nicotinate ribonucleoside	3-methylglutaconate	vanillactate
p-cresol sulfate	methyl glucopyranoside (alpha + beta)	androstenediol (3beta,17beta) disulfate (2)
androstenediol (3alpha, 17alpha) monosulfate (3)	ergothioneine	N-palmitoyl-sphinganine (d18:0/16:0)
hydroxyasparagine**	16-hydroxypalmitate	guaiacol sulfate
2-oxoarginine*	1-adrenoyl-GPC (22:4)*	arachidonoylcholine
1-methylguanidine	pyridoxate	phenylalanylphenylalanine
methylmalonate (MMA)	pyrraline	5alpha-androstan-3beta,17alpha-diol disulfate
3-methyl catechol sulfate (1)	1-palmitoyl-2-meadoyl-GPC (16:0/20:3n9)*	N-formylanthranilic acid
glucuronate	N-acetylglutamate	glyco-beta-muricholate**
2-arachidonoyl-GPE* (20:4)*	N-acetyl-aspartyl-glutamate (NAAG)	cysteinylglycine disulfide*
3-hydroxyoleate*	21-hydroxypregnenolone disulfate	1-arachidonoyl-GPC (20:4n6)*
12,13-DiHOME	N-acetyl-2-aminooctanoate*	1-methyl-4-imidazoleacetate
sphingomyelin (d18:1/24:1, d18:2/24:0)*	1-palmitoyl-2-linoleoyl-GPC (16:0/18:2)	N-palmitoyl-sphingosine (d18:1/16:0)
arachidoylcarnitine (C20)*	arabitol/xylitol	glycosyl-N-(2-hydroxynervonoyl)-sphingosine(d18:1/24:1(2OH))*
caproate (6:0)	pseudouridine	1-palmityl-2-arachidonoyl-GPC (O-16:0/20:4)*
corticosterone	imidazole lactate	hypoxanthine
glycosyl-N-stearoyl-sphingosine (d18:1/18:0)	2-hydroxyarachidate*	sphingomyelin (d18:1/21:0, d17:1/22:0, d16:1/23:0)*
4-imidazoleacetate	betonicine	2-hydroxybehenate
cysteine sulfinic acid	laurylcarnitine (C12)	eicosenoate (20:1)
anthranilate	sphingosine	N1-methyladenosine
5-bromotryptophan	2-aminophenol sulfate	pro-hydroxy-pro
3-methylhistidine	iminodiacetate (IDA)	2-linoleoylglycerol (18:2)
oxalate (ethanedioate)	carnitine	gentisate
2,3-dihydroxyisovalerate	vanillylmandelate (VMA)	2-methylmalonyl carnitine
gamma-glutamylisoleucine*	gluconate	cholesterol
cis-aconitate	bilirubin (Z,Z)	methylnaphthyl sulfate (2)*
gamma-glutamylalanine	1-linoleoyl-GPI (18:2)*	N-alpha-acetylornithine
docosatrienoate (22:3n3)	glycoursodeoxycholate	ethylmalonate

linoleoylcarnitine (C18:2)*	methylsuccinate	epiandrosterone sulfate
2-linoleoyl-GPE* (18:2)*	9,10-DiHOME	N1-Methyl-2-pyridone-5-carboxamide
4-hydroxyglutamate	C-glycosyltryptophan	etiocholanolone glucuronide
trimethylamine N-oxide	2-aminoheptanoate	myristoleoylcarnitine (C14:1)*
retinal	xanthine	sphingomyelin (d18:1/22:1, d18:2/22:0, d16:1/24:1)*
tetradecanedioate (C14-DC)	5-hydroxyhexanoate	nervonoylcarnitine (C24:1)*
sphingomyelin (d18:1/14:0, d16:1/16:0)*	phytanate	trans-4-hydroxyproline
malonate	2-piperidinone	2-oleoyl-GPC* (18:1)*
N-acetylproline	O-sulfo-L-tyrosine	bilirubin (E,E)*
gamma-glutamylvaline	1-oleoyl-2-docosapentaenoyl-GPC (18:1/22:5n3)*	hydantoin-5-propionic acid
S-methylmethionine	phenylpyruvate	fumarate
4-hydroxyphenylpyruvate	glutarylcarnitine (C5-DC)	3-carboxy-4-methyl-5-pentyl-2-furanpropionate (3-Cmpfp)**
gamma-glutamylleucine	gamma-glutamylglutamate	myo-inositol
sphingomyelin (d17:2/16:0, d18:2/15:0)*	N-acetylarginine	beta-hydroxyisovalerate
quinolinate	1,7-dimethylurate	acetylcarnitine (C2)
glycosyl ceramide (d18:1/20:0, d16:1/22:0)*	N-acetylglutamine	phenylacetylcarnitine
6-hydroxyindole sulfate	4-methylcatechol sulfate	21-hydroxypregnenolone monosulfate (1)
glutarate (C5-DC)	N-oleoyltaurine	palmitoleoylcarnitine (C16:1)*
4-allylphenol sulfate	oleoylcarnitine (C18:1)	margaroylcarnitine (C17)*
3-hydroxydecanoate	andro steroid monosulfate C19H28O6S (1)*	2-hydroxyglutarate
isoursodeoxycholate	decanoylcarnitine (C10)	3-indoxyl sulfate
urea	1-(1-enyl-stearoyl)-2-docosahexaenoyl-GPE (P-18:0/22:6)*	heptanoate (7:0)
myristoyl dihydrosphingomyelin (d18:0/14:0)*	stearoyl sphingomyelin (d18:1/18:0)	linoleoylcholine*
inosine	3-methylcytidine	N2,N2-dimethylguanosine
glycosyl-N-behenoyl-sphingadienine (d18:2/22:0)*	palmitoyl ethanolamide	glycocholenate sulfate*
3-hydroxybutyrylcarnitine (1)	linolenoylcarnitine (C18:3)*	tiglylcarnitine (C5:1-DC)
bilirubin (E,Z or Z,E)*	5-methylthioadenosine (MTA)	1-palmitoleoyl-GPI* (16:1)*
cys-gly, oxidized	O-acetylhomoserine	N-carbamoylalanine

Supplementary Table S4. Associations of *GCKR* rs780094-T with amino acids, carbohydrates and other metabolites

Pathway	Beta	*P** value	*P*** value	Novel
**Amino acids**				
Alanine	0.080	7.77E-04	0.013	No
Serine	-0.065	1.58E-03	4.4E-4	Yes
Threonine	-0.094	1.36E-11	1.3E-4	Yes
Tryptophan pathway:				
Indolelactate	0.066	4.23E-04	0.126	Yes
N-acetyltryptophan	0.066	8.78E-03	0.033	Yes
Valine pathway:				
3-aminoisobutyrate	-0.082	3,76E-07	0.025	Yes
**Carbohydrates**				
Lactate	0.066	1.33E-02	0.012	No
Mannose	-0.325	3.58E-127	1.03E-85	No
Pyruvate	0.083	1.25E-04	2E-5	No
**Other metabolites**				
Gamma-glutamylthreonine	-0.065	2.08E-07	0.505	Yes
Taurocholenate sulfate	-0.063	2.66E-06	0.801	Yes
Retinol (Vitamin A)	0.063	0.022	0.178	Yes
3-hydroxybutyrate	-0.057	0.019	2.9E-4	No

*P** values were adjusted for batch effect, age and total triglycerides. *P*** values are adjusted for batch effect, age, total triglycerides, fasting glucose, and with all metabolites (N=46) showing significant association with rs780094-T of *GCKR*.

Supplementary Table S5. Association of *GCKR* rs780094-T with lipids

Pathway	Beta	*P** value	*P*** value	Sub class	Direct parent	Novel
**Glycerolipids**						
Triacylglycerides*	0.107	-	0.484	TAG	TAG	No
Palmitoleoyl-linoleoyl-glycerol (16:1/18:2) (1)**	0.109	0.260	0.108	DAG	1,2-DAG	Yes
Myristoyl-linoleoyl-glycerol (14:0/18:2) (1)**	0.120	0.017	0.585	DAG	1,2-DAG	Yes
Palmitoyl-linoleoyl-glycerol (16:0/18:2) (2)**	0.057	0.447	0.463	DAG	1,2-DAG	Yes
Oleoyl-linoleoyl-glycerol (18:1/18:2) (1)**	0.065	0.088	0.387	DAG	1,2-DAG	Yes
Oleoyl-linoleoyl-glycerol (18:1/18:2) (2)**	0.061	0.056	0.560	DAG	1,2-DAG	Yes
DAG (12:0/18:1, 14:0/16:1, 16:0/14:1) (2)**	0.091	0.088	0.391	DAG	1,2-DAG	Yes
Oleoyl-arachidonoyl-glycerol (18:1/20:4) (1)**	0.095	0.314	0.288	DAG	1,2-DAG	Yes
Oleoyl-arachidonoyl-glycerol (18:1/20:4) (2)**	0.087	0.216	0.428	DAG	1,2-DAG	Yes
Oleoyl-oleoyl-glycerol (18:1/18:1) (2)**	0.087	0.523	0.065	DAG	1,2-DAG	Yes
1-palmitoleoylglycerol (16:1)	0.082	0.750	0.758	MAG	1-MAG	Yes
1-oleoylglycerol (18:1)	0.057	0.515	0.011	MAG	1-MAG	Yes
1-myristoylglycerol (14:0)	0.073	0.096	0.795	MAG	1-MAG	Yes
**Glycerophospholipids**						
1-stearoyl-2-arachidonoyl-GPI (18:0/20:4)	0.058	0.312	0.05	GPI	PI	Yes
1-palmitoyl-2-oleoyl-GPE (16:0/18:1)	0.085	0.114	0.005	GPE	PE	Yes
1-palmitoyl-2-docosahexaenoyl-GPE (16:0/22:6)	0.087	0.047	6.7E-4	GPE	PE	Yes
1-stearoyl-2-docosahexaenoyl-GPE (18:0/22:6)	0.098	0.026	4.3E-4	GPE	PE	Yes
1-palmitoyl-2-linoleoyl-GPE (16:0/18:2)	0.063	0.708	0.265	GPE	PE	Yes
1-stearoyl-2-oleoyl-GPE (18:0/18:1)	0.075	0.589	0.991	GPE	PE	Yes
1-stearoyl-2-linoleoyl-GPE (18:0/18:2)	0.07	0.823	0.325	GPE	PE	Yes
1-stearoyl-2-arachidonoyl-GPE (18:0/20:4)	0.064	0.669	0.373	GPE	PE	Yes
1-oleoyl-2-docosahexaenoyl-GPE (18:1/22:6)	0.082	0.026	0.797	GPE	PE	Yes
1-oleoyl-2-arachidonoyl-GPE (18:1/20:4)	0.078	0.086	0.873	GPE	PE	Yes
1-stearoyl-GPE (18:0)	0.077	0.011	0.396	GPE	Lyso-PEth	Yes
1-palmitoyl-GPE (16:0)	0.068	0.010	0.998	GPE	Lyso-PEth	Yes
1-myristoyl-2-arachidonoyl-GPC (14:0/20:4)	0.092	0.002	0.065	GPC	PC	Yes
1-myristoyl-2-palmitoyl-GPC (14:0/16:0)	0.065	0.020	0.054	GPC	PC	Yes
1-palmitoyl-2-palmitoleoyl-GPC (16:0/16:1)	0.064	0.139	0.323	GPC	PC	Yes
1-palmitoleoyl-GPC(16:1)	0.065	0.021	0.813	GPC	Lyso-PCho	Yes
1-(1-enyl-palmitoyl)-2-palmitoleoyl-GPC (P-16:0/16:1)	-0.070	0.005	0.051	GPC	PlCho	Yes
1-(1-enyl-palmitoyl)-2-linoleoyl-GPC (P-16:0/18:2)	-0.069	0.057	0.068	GPC	PlCho	Yes
1-(1-enyl-palmitoyl)-2-oleoyl-GPC (P-16:0/18:1)	-0.063	0.125	0.280	GPC	PlCho	Yes
**Sphingolipids**						
lactosyl-N-nervonoyl-sphingosine (d18:1/24:1)	-0.081	0.008	0.192	GSL	Lactosylceramide	Yes
lactosyl-N-palmitoyl-sphingosine (d18:1/16:0)	-0.061	0.004	0.29	GSL	Lactosylceramide	Yes

Abbreviations: DAG, Diacylglycerol; GPC, Glycerophosphocoline; GPE, Glycerophosphoethanolamine; GPI, Glycerophosphoinositol; GSL, Glycerosphingolipid; Lyso-PCho, Lysophosphatidylcholine; Lyso-PEth, Lysophosphatidylethanolamine; MAG, Monoacylglycerol; PC, Phosphatidylcholine; PE, Phosphatidylethanolamine; PI, Phosphatidylinositol; PlCho, Plasmalogen-Choline; TAG, Triacylglycerol.

*TAG was measured using an enzymatic method. **The numbers (1) and (2) in parentheses refer to different stereoisomers of the metabolites. *P** values were adjusted for batch effect, age, fasting glucose and triglycerides. *P*** values are adjusted for batch effect, age, total triglycerides, fasting glucose and with all metabolites (N=46) showing significant association with rs780094-T of *GCKR*.

Supplementary Table S6. Association of amino acids, carbohydrates and other metabolites with Matsuda ISI, BMI and ALT

Metabolite	Matsuda	BMI	ALT
	**Beta**	**Beta**	**Beta**
**Amino acids**			
Alanine	-0,339***	0,178***	0,145***
Serine	0,151***	-0,132***	-0,098***
Threonine	-0,028*	-0,031*	0,004
Tryptophan pathway:			
Indolelactate	-0,156***	0,076***	0,088***
N-acetyltryptophan	-0,281***	0,233***	0,188***
Valine pathway:			
3-aminoisobutyrate	0,118***	-0,056***	-0,013
**Carbohydrates**			
Lactate	-0,328***	0,137***	0,127***
Mannose	-0,273***	0,303***	0,138***
Pyruvate	-0,343***	0,171***	0,105***
**Other metabolites**			
Gamma-glutamylthreonine	-0,082***	0,018	0,076***
Taurocholenate sulfate	-0,131***	0,087***	0,155***
Retinol (Vitamin A)	-0,118***	0,063***	0,127***
3-hydroxybutyrate (BHBA)	0,182***	-0,077***	0,009***

*P<0.05. **P<0.01. ***P<0.001. Beta and P values were obtained from linear regression, adjusted for batch and age.

Supplementary Table S7. Association of lipids with Matsuda ISI, BMI and ALT

Lipids	Matsuda	BMI	ALT
**Glycerolipids**	**Beta**	**Beta**	**Beta**
Triacylglycerides*	-0,477***	0,315***	0,204***
palmitoleoyl-linoleoyl-glycerol (16:1/18:2) (1)	-0,321***	0,216***	0,095***
myristoyl-linoleoyl-glycerol (14:0/18:2) (1)	-0,362***	0,194***	0,108***
palmitoyl-linoleoyl-glycerol (16:0/18:2) (2)	-0,270***	0,183***	0,098***
oleoyl-linoleoyl-glycerol (18:1/18:2) (1)	-0,266***	0,155***	0,050*
oleoyl-linoleoyl-glycerol (18:1/18:2) (2)	-0,259***	0,155***	0,052*
diacylglycerol (12:0/18:1, 14:0/16:1, 16:0/14:1) (2)	-0,282***	0,163***	0,128***
oleoyl-arachidonoyl-glycerol (18:1/20:4) (1)	-0,274***	0,206***	0,145***
oleoyl-arachidonoyl-glycerol (18:1/20:4) (2)	-0,233***	0,183***	0,117***
oleoyl-oleoyl-glycerol (18:1/18:1) (2)	-0,304***	0,221***	0,115***
1-palmitoleoylglycerol (16:1)	-0,258***	0,196***	0,158***
1-oleoylglycerol (18:1)	-0,194***	0,123***	0,109***
1-myristoylglycerol (14:0)	-0,212***	0,128***	0,121***
**Glycerophospholipids**			
1-stearoyl-2-arachidonoyl-GPI (18:0/20:4)	-0,150***	0,141***	0,092***
1-palmitoyl-2-oleoyl-GPE (16:0/18:1)	-0,248***	0,167***	0,127***
1-palmitoyl-2-docosahexaenoyl-GPE (16:0/22:6)	-0,104***	0,080***	0,103***
1-stearoyl-2-docosahexaenoyl-GPE (18:0/22:6)	-0,279***	0,229***	0,192***
1-palmitoyl-2-linoleoyl-GPE (16:0/18:2)	-0,087***	0,017	-0,006
1-stearoyl-2-oleoyl-GPE (18:0/18:1)	-0,313***	0,189***	0,158***
1-stearoyl-2-linoleoyl-GPE (18:0/18:2)	-0,206***	0,104***	0,049***
1-stearoyl-2-arachidonoyl-GPE (18:0/20:4)	-0,190***	0,151***	0,091***
1-oleoyl-2-docosahexaenoyl-GPE (18:1/22:6)	0,170***	-0,136***	-0,054**
1-oleoyl-2-arachidonoyl-GPE (18:1/20:4)	-0,053**	-0,002	0,023
1-stearoyl-GPE (18:0)	0,057***	-0,086***	-0,047***
1-palmitoyl-GPE (16:0)	0,156***	-0,164***	-0,101***
1-myristoyl-2-arachidonoyl-GPC (14:0/20:4)	-0,240***	0,145***	0,124***
1-myristoyl-2-palmitoyl-GPC (14:0/16:0)	-0,134***	0,046**	0,075***
1-palmitoyl-2-palmitoleoyl-GPC (16:0/16:1)	-0,225***	0,192***	0,176***
1-palmitoleoyl-GPC (16:1)	-0,029*	0,037**	0,070***
1-(1-enyl-palmitoyl)-2-palmitoleoyl-GPC (P-16:0/16:1)	0,292***	-0,101***	-0,079**
1-(1-enyl-palmitoyl)-2-linoleoyl-GPC (P-16:0/18:2)	0,420***	-0,294***	-0,196***
1-(1-enyl-palmitoyl)-2-oleoyl-GPC (P-16:0/18:1)	0,410***	-0,240***	-0,168***
**Sphingolipids**			
lactosyl-N-nervonoyl-sphingosine (d18:1/24:1)	0,276***	-0,216***	-0,145***
lactosyl-N-palmitoyl-sphingosine (d18:1/16:0)	0,236***	-0,184***	-0,152***

**P*=<0.05. ***P*=<0.01. ****P*=<0.001. Beta and *P* values were obtained from linear regression, adjusted for batch and age.

Supplementary Table S8. Association of amino acids, carbohydrates and other metabolites with Matsuda ISI, BMI and ALT

**Metabolite**	**Matsuda**	**BMI**	**ALT**	**Matsuda**	**BMI**	**ALT**
	**Beta (T-allele)**	**Beta (T-allele)**	**Beta (T-allele)**	**Beta (CC)**	**Beta (CC)**	**Beta (CC)**
**Amino acids**						
Alanine	-0,340***	0,170***	0,147***	-0,360***	0,194***	0,196***
Serine	0,159***	-0,139***	-0,095***	0,086*	-0,085*	-0,130***
Threonine						
Tryptophan pathway:	-0,029	-0,029	0,011	-0,005	-0,073*	-0,026
Indolelactate	-0,152***	0,068***	0,098***	-0,203***	0,116**	0,058
N-acetyltryptophan						
Valine pathway:	-0,288***	0,236***	0,202***	-0,255***	0,213***	0,186***
3-aminoisobutyrate						
**Carbohydrates**	0,111***	-0,046**	-0,015	0,161***	-0,104**	0,008
Lactate	-0,322***	0,134***	0,126***	-0,383***	0,151***	0,175***
Mannose	-0,276***	0,324***	0,147***	-0,262***	0,269***	0,171***
Pyruvate						
**Other metabolites**	-0,336***	0,173***	0,089***	-0,426***	0,163***	0,226***
Gamma-glutamylthreonine	-0,089***	0,021	0,075***	-0,024	-0,026	0,118**
Taurocholenate sulfate	-0,137***	0,098***	0,170***	-0,065	0,027	0,131***
Retinol (Vitamin A)	-0,119***	0,070***	0,129***	-0,126***	0,015	0,137***
3-hydroxybutyrate (BHBA)	0,200***	-0,078***	0,002	0,073*	-0,059	0,055

*P<0.05. **P<0.01. ***P<0.001. Beta and P values were obtained from linear regression, adjusted for batch and age.

Supplementary Table S9. Association of lipids with Matsuda ISI, BMI and ALT						
**Lipids**	**Matsuda**	**BMI**	**ALT**	**Matsuda**	**BMI**	**ALT**
**Glycerolipids**	**β (T-allele)**	**β (T-allele)**	**β (T-allele)**	**β (CC)**	**β (CC)**	**β (CC)**
Triacylglycerides*	-0,476***	0,317***	0,206***	-0,489***	0,298***	0,242***
palmitoleoyl-linoleoyl-glycerol (16:1/18:2) (1)	-0,322***	0,223***	0,091***	-0,351***	0,185***	0,141**
myristoyl-linoleoyl-glycerol (14:0/18:2) (1)	-0,364***	0,204***	0,111***	-0,387***	0,145**	0,137**
palmitoyl-linoleoyl-glycerol (16:0/18:2) (2)	-0,263***	0,181***	0,096***	-0,321***	0,205***	0,152***
oleoyl-linoleoyl-glycerol (18:1/18:2) (1)	-0,264***	0,159***	0,043**	-0,292***	0,145***	0,088*
oleoyl-linoleoyl-glycerol (18:1/18:2) (2)	-0,256***	0,159***	0,047**	-0,292***	0,153***	0,092*
DAG (12:0/18:1, 14:0/16:1, 16:0/14:1) (2)	-0,278***	0,159***	0,135***	-0,253***	0,142**	0,107*
oleoyl-arachidonoyl-glycerol (18:1/20:4) (1)	-0,264***	0,201***	0,139***	-0,359***	0,231***	0,237***
oleoyl-arachidonoyl-glycerol (18:1/20:4) (2)	-0,228***	0,176***	0,106***	-0,291***	0,229***	0,230***
oleoyl-oleoyl-glycerol (18:1/18:1) (2)	-0,298***	0,225***	0,104***	-0,350***	0,203***	0,199***
1-palmitoleoylglycerol (16:1)	-0,266***	0,195***	0,161***	-0,242***	0,207***	0,173***
1-oleoylglycerol (18:1)	-0,192***	0,126***	0,110***	-0,209***	0,116**	0,119**
1-myristoylglycerol (14:0)	-0,218***	0,126***	0,122***	-0,195***	0,135***	0,161***
**Glycerophospholipids**						
1-stearoyl-2-arachidonoyl-GPI (18:0/20:4)	-0,131***	0,139***	0,079***	-0,256***	0,168***	0,192***
1-palmitoyl-2-oleoyl-GPE (16:0/18:1)	-0,235***	0,167***	0,122***	-0,310***	0,171***	0,179***
1-palmitoyl-2-docosahexaenoyl-GPE (16:0/22:6)	-0,093***	0,075***	0,100***	-0,179***	0,109**	0,132
1-stearoyl-2-docosahexaenoyl-GPE (18:0/22:6)	-0,270***	0,218***	0,190***	-0,350***	0,286***	0,245***
1-palmitoyl-2-linoleoyl-GPE (16:0/18:2)	-0,076***	0,017	-0,013	-0,144***	0,016	0,008
1-stearoyl-2-oleoyl-GPE (18:0/18:1)	-0,299***	0,187***	0,150***	-0,383***	0,207***	0,237***
1-stearoyl-2-linoleoyl-GPE (18:0/18:2)	-0,193***	0,103***	0,038*	-0,268***	0,109**	0,100**
1-stearoyl-2-arachidonoyl-GPE (18:0/20:4)	-0,173***	0,146***	0,078***	-0,265***	0,167***	0,174***
1-oleoyl-2-docosahexaenoyl-GPE (18:1/22:6)	-0,046*	0,012	0,049*	-0,003	0,001	0,035
1-oleoyl-2-arachidonoyl-GPE (18:1/20:4)	-0,046*	-0,004	0,019	-0,086	0,012***	0,053
1-stearoyl-GPE (18:0)	0,070***	-0,092***	-0,066***	-0,017	-0,051***	0,021
1-palmitoyl-GPE (16:0)	0,167***	-0,163***	-0,110***	0,088*	-0,175	-0,100**
1-myristoyl-2-arachidonoyl-GPC (14:0/20:4)	-0,236***	0,139***	0,122***	-0,267***	0,164***	0,170***
1-myristoyl-2-palmitoyl-GPC (14:0/16:0)	-0,134***	0,041*	0,076***	-0,143***	0,058	0,102*
1-palmitoyl-2-palmitoleoyl-GPC (16:0/16:1)	-0,223***	0,195***	0,184***	-0,243***	0,164*	0,189***
1-palmitoleoyl-GPC (16:1)	-0,028	0,037*	0,073***	-0,046	0,032***	0,070
1-(1-enyl-palmitoyl)-2-palmitoleoyl-GPC (P-16:0/16:1)	0,299***	-0,105***	-0,083***	0,263***	-0,080***	-0,081*
1-enyl-palmitoyl)-2-linoleoyl-GPC (P-16:0/18:2)	0,428***	-0,291***	-0,198***	0,391***	-0,312***	-0,245***

1-(1-enyl-palmitoyl)-2-oleoyl-GPC (P-16:0/18:1)	0,417***	-0,239***	-0,173***	0,384***	-0,239***	-0,201***
**Sphingolipids**						
lactosyl-N-nervonoyl-sphingosine (d18:1/24:1)	0,284***	-0,225***	-0,152***	0,258***	-0,172	-0,151**
lactosyl-N-palmitoyl-sphingosine (d18:1/16:0)	0,233***	-0,179***	-0,160***	0,268***	-0,213	-0,153***

*P=<0.05. **P=<0.01. ***P=<0.001. Beta and P values were obtained from linear regression, adjusted for batch and age.
